# Effect of anti-inflammatory diets on health-related quality of life in adults with chronic disease: a systematic review and meta-analysis

**DOI:** 10.1136/bmjnph-2025-001257

**Published:** 2025-06-10

**Authors:** Lynette Law, Joshua J Heerey, Brooke L Devlin, Peter Brukner, Alysha M De Livera, Joanne Kemp, Amanda Attanayake, Søren Thorgaard Skou, Alessio Bricca, Adam G Culvenor

**Affiliations:** 1 La Trobe Sport and Exercise Medicine Research Centre, School of Allied Health, Human Services and Sport, La Trobe University, Melbourne, Victoria, Australia; 2 School of Human Movement and Nutrition Sciences, The University of Queensland - St Lucia Campus, Brisbane, Queensland, Australia; 3 Department of Mathematics and Statistics, La Trobe University, Melbourne, Victoria, Australia; 4 Center for Muscle and Joint Health, Department of Sports Science and Clinical Biomechanics, University of Southern Denmark, Odense, Denmark; 5 The Research and Implementation Unit PROgrez, Department of Physiotherapy and Occupational Therapy, Næstved-Slagelse-Ringsted Hospitals, Slagelse, Denmark

**Keywords:** Precision nutrition, Musculo-skeletal health, Dietary patterns, Nutritional treatment, Nutrition assessment

## Abstract

**Objective:**

To evaluate the effectiveness of anti-inflammatory diets on health-related quality of life (HRQOL) in adults with at least one chronic disease.

**Design:**

Systematic review and meta-analysis of randomised controlled trials (RCTs).

**Data sources:**

MEDLINE, EMBASE, CINAHL, Web of Science and the Cochrane Centre Register of Controlled Trials from inception to 6 May 2024.

**Eligibility criteria for selecting studies:**

Full-text RCTs published in English assessing the effectiveness of any anti-inflammatory dietary intervention (ie, a diet that emphasises the intake of nutrient-rich, minimally processed foods rich in polyphenols, carotenoids and omega-3 polyunsaturated fatty acids and limits highly-processed, pro-inflammatory foods) on HRQOL in adults with at least one chronic disease were included.

**Methods:**

Data extraction, risk-of-bias assessments and strength-of-evidence assessments were done by two independent reviewers. Pooled effects (standardised mean difference (SMD)) for HRQOL (separated into mental and physical component scores wherever possible) were calculated using random effects models with restricted maximum likelihood estimations. Subgroup analyses and meta-regressions were performed to assess the influence of study-level characteristics on HRQOL outcomes.

**Results:**

23 studies reporting HRQOL for 3294 participants were included. The most common chronic diseases were type two diabetes, musculoskeletal conditions and cardiovascular conditions. Anti-inflammatory diets were associated with small improvements in HRQOL physical component scores compared with usual care/other dietary interventions (18 trials, SMD=0.17, 95% CI 0.06 to 0.27) but not in mental component scores (18 trials, SMD=0.09, 95% CI −0.02 to 0.20) or general HRQOL scores (four trials, SMD=0.27, 95% CI −0.22 to 0.77). Pooled effects did not differ by available study-level characteristics; however, diet-only interventions (compared with multi-component interventions) had a greater effect on mental component scores. No study met the Cochrane criteria for low risk of bias. Certainty of evidence was low for physical and mental HRQOL scores and very low for general HRQOL scores.

**Conclusions:**

In adults with at least one chronic disease, anti-inflammatory diets lead to small improvements in physical component HRQOL, which may not be clinically relevant. No effect was found on the mental component or general HRQOL. Further high-quality RCTs may change this conclusion.

**WHAT IS ALREADY KNOWN ON THIS TOPIC**

- An increasing proportion of older adults have at least one chronic disease, which is associated with increased healthcare utilisation, polypharmacy and compromised quality of life.
- Health-related quality of life (HRQOL) measures provide data that can be compared across different types and numbers of chronic diseases (eg, multimorbidity) compared with disease-specific measures, which can be applied only to one type of disease.
- Anti-inflammatory diets are recognised for their ability to improve disease-specific risk factors and biomarkers across diseases, but the effect of these anti-inflammatory diets on HRQOL in individuals with chronic disease is unclear.

**WHAT THIS STUDY ADDS**

- Anti-inflammatory diets (eg, low-carbohydrate, Mediterranean) may lead to small improvements in the physical component of HRQOL compared with usual care or other diets in patients with more than one chronic disease, but the effect size is small, of low certainty and may not be clinically relevant.
- Anti-inflammatory diets provide no additional benefit over usual care or other diets for mental component or general HRQOL.
- The importance of further high-quality trials powered to detect clinically relevant effects of anti-inflammatory diets, along with improved reporting of dietary intervention details and participant adherence, is emphasised.

**HOW THIS STUDY MIGHT AFFECT RESEARCH, PRACTICE OR POLICY**

- Clinicians may recommend anti-inflammatory dietary strategies as part of chronic disease care without negatively affecting patients' mental well-being.
- The variability in how anti-inflammatory diets are defined highlights the need for clearer definitions to support consistent implementation and evidence-based dietary recommendations

## Introduction

 Chronic diseases are the leading cause of disability and premature death worldwide.^1^ Over half of all deaths are attributable to chronic diseases such as ischaemic heart disease, stroke, diabetes mellitus, and chronic kidney disease.^2^ The coexistence of two or more conditions in an individual, known as multimorbidity, is a growing global health priority due to its substantial impact on quality of life, physical and mental decline, and increased healthcare utilisation.^3 4^ The mechanisms underlying the rising prevalence of chronic disease are complex and interrelated, driven largely by changes in lifestyle factors, ageing and broad socioeconomic status.^5^

Health-related quality of life (HRQOL) is an established concept and core outcome in chronic disease research, where the focus is on improving overall perceived health and well-being rather than simply prolonging life.^6 7^ Several validated questionnaires have been developed and tested for assessing HRQOL across different clinical settings and populations. Disease-specific instruments, as the name suggests, measure unique elements of a particular disease that may not apply to others, whereas generic HRQOL instruments capture broader dimentions of health and enable the comparison of disease burden and intervention effects across different types or combinations of chronic diseases.^5^ The ability to make such comparisons supports the development of patient-centred interventions that are effective not only in managing individual diseases but also in addressing multimorbidity.

Many chronic diseases are linked to modifiable lifestyle risk factors and are responsive to lifestyle interventions such as exercise and diet.^8^,^11^ The relationship between diet and inflammation is well-established.^12^,^15^ Beyond conventional weight loss strategies, dietary approaches that are anti-inflammatory aim to mitigate the inflammatory processes underlying chronic disease development by emphasising minimally processed and nutrient-dense foods rich in polyphenols, carotenoids, fibre, monounsaturated and omega-3 polyunsaturated fatty acids^16 17^. These diets also limnit foods believed to promote low-grade systemic inflammation (eg, refined carbohydrates, added sugar, seed oils). Examples of anti-inflammatory dietary patterns are the Mediterranean, low-carbohydrate (eg, ketogenic) and Nordic diets and Dietary Approaches to Stop Hypertension (DASH), which have been associated with reduced levels of inflammation.^18^,^21^

Research suggests that anti-inflammatory diets may help improve disease-specific outcomes, including in cardiovascular disease,^22 23^ diabetes,^24^ arthritis^25^ and depression.^26 27^ However, their influence on generic health outcomes, such as HRQOL, which applies across diseases, has yielded mixed results.^28 29^ Therefore, the objective of our systematic review is to assess the effectiveness of anti-inflammatory diets on HRQOL in adults with one or more chronic diseases.

## Methods

The current systematic review was performed following the Cochrane handbook recommendations.^30^ It is reported according to Preferred Reporting Items for Systematic reviews and Meta-Analysis guidelines^31^ and was prospectively registered in Open Science Framework (https://osf.io/f5h86/).

### Eligibility criteria

Full-text randomised controlled trials (RCTs) published in English assessing the effectiveness of any anti-inflammatory dietary intervention on HRQOL in adults with at least one chronic disease were included. We included RCTs as they provide the highest level of evidence by minimising bias through randomisation, allowing for reliable comparisons of intervention effectiveness. We defined chronic disease in accordance with the WHO criteria: “a noncommunicable condition that is long in duration, typically has a slow progression, and which requires ongoing medical attention or is associated with functional impairment or disability”.^32^ Acknowledging that no universal definition exists for anti-inflammatory diets, we included studies that described their diets as ‘anti-inflammatory’ rather than applying a pre-defined dietary framework. This approach allowed for the inclusion of all interventions in which authors aimed to evaluate the effects of what they considered an anti-inflammatory diet. Anti-inflammatory dietary interventions typically promote foods/drinks known to decrease systemic inflammation (often low-carbohydrate, nutrient-dense, whole foods rich in fibre, antioxidants and monounsaturated/omega-3 polyunsaturated fats) and which exclude foods/drinks known to promote inflammation (eg, refined white carbohydrates, seed oils).^12 16 33^ No restrictions were placed on comparator group interventions, reflecting the diverse real-world contexts in which these diets are studied and implemented, and heterogeneity across studies was examined in the meta-analysis. We defined HRQOL as a multidimensional assessment of how a disease or treatment affects an individual’s self-perceived physical, mental and/or emotional well-being. HRQOL can be assessed using generic (ie, non-disease specific) measures, such as the Medical Outcomes Study 36-item Short-Form Health Survey (SF-36),^34^ SF-12^35^ and EQ-5D.^36^

### Data sources and study selection

We searched MEDLINE, EMBASE, the Cumulative Index to Nursing and Allied Health Literature, Web of Science and the Cochrane Central Register of Controlled Trials databases for relevant RCTs published in English from inception to 6 May 2024 (online supplemental eMethods 1 details the search strategy). We also searched for eligible trials from trial registries, personal communications, books and theses or dissertations. The WHO International Clinical Trials Registry Platform was also searched for trials that were registered as complete. Reference lists of all publications considered for inclusion were hand-searched recursively until no additional eligible publications were identified. Two team members (LL, AA) independently assessed all titles and abstracts of identified reports for eligibility. Following this, we reviewed full texts, and if required data could not be extracted from the published studies, we requested the required data for meta-analysis from corresponding authors. If no response was received after two attempts and the study had no appropriate data available for extraction or imputation,^37^ the study was excluded.

### HRQOL Outcomes

HRQOL is commonly assessed with a variety of patient-reported non-disease-specific tools. One of the most common is the SF-36,^34^ which is scored across eight subscales. These subscales are typically combined to form two higher-order summary scores: the Physical Component Score (PCS) and Mental Component Score (MCS).^38^ Variants of the SF-36 include the RAND-36, 20-item (SF-20)^39^ and 12-item Short-Form surveys (SF-12).^40^ Other common HRQOL tools, which reflect a general HRQOL score, include the EQ-5D,^41^ Assessment of Quality of Life-8 Dimension (AQoL-8d),^42^ US Centre for Disease Control and Prevention (CDC) Healthy Days^43^ and WHO Quality of Life Assessment (WHOQOL).^7^

### Data extraction

The following information was independently extracted from included studies by two authors (LL, JJH): sample size, primary chronic condition, participant characteristics (ie, mean age, proportion female, body mass index), intervention and comparator group details (eg, frequency of support sessions, delivery mode, duration, co-interventions), duration of follow-up, HRQOL tool used and method of dietary assessment. When several intervention groups were included in a trial, the between-group difference was reported for either the anti-inflammatory dietary intervention and usual care group; or the combination group including diet (eg, diet and exercise) and the non-dietary intervention group, in line with the Cochrane handbook recommendation on how to handle multiple-arm studies.^44^ Discrepancies were resolved by consensus or consultation with a third reviewer (AGC).

### Risk of bias assessment

Two authors (LL and either BLD or JH) independently assessed the risk of bias of included trials using the Cochrane Collaboration tool 2.0.^45^ The trials were graded (low, some concerns, high) based on sequence generation, allocation concealment, blinding of outcome assessor, incomplete outcome data and selective outcome reporting. Discrepancies were resolved by a third reviewer (AGC), as previously described.^46 47^

### Dietary intervention assessment

As pre-specified in our protocol, based on a combination of theoretical and clinical considerations, two authors (LL, BLD) independently assessed the overall anti-inflammatory potential of dietary interventions in the included studies as adequate, inadequate or unable to determine. This score was based on the potential of an intervention to mitigate systemic inflammation and suppress pro-inflammatory responses according to three criteria: (i) pro-inflammatory foods and nutrients excluded, (ii) anti-inflammatory foods and nutrients included and (iii) recommended carbohydrate content of diets. While there is no single universal definition of an anti-inflammatory diet, important beneficial characteristics, such as a high intake of minimally processed foods rich in nutrients such as polyphenols, fibre, polyunsaturated and monounsaturated fatty acids, have been associated with a higher likelihood of an anti-inflammatory effect.^12 33 48 49^

### Data synthesis and analysis

Pooling of study-specific estimates of HRQOL outcomes was completed using random-effects meta-analysis and restricted maximum likelihood models due to the expected heterogeneity in participants, interventions and comparator characteristics. Standardised mean differences (SMD) (for the difference between the intervention group (IG) and control group (CG) change score) and 95% CI were calculated as HRQOL was reported in the included studies with different outcome measures. The magnitude of the effect size of the pooled SMD was interpreted as 0.2 representing a small effect, 0.5 a moderate effect and 0.8 a large effect. SMDs of 0.5 or larger were considered clinically relevant.^50^

If the required data to calculate SMD and its variance was not available in the published paper and was not provided by the authors on request, we used recommendations from the Cochrane handbook to derive missing data where possible.^37^ Detailed study-specific approaches to calculate the SMD and its variance are described in online supplemental eMethods 2. Most HRQOL instruments assessed physical and mental aspects of HRQOL separately (eg, SF-36, SF-12). This enabled (pre-specified) separate data pooling of the two composite scores: PCS and MCS. Studies providing a general HRQOL score were pooled separately, consistent with previous approaches pooling HRQOL data.^51^ Statistical heterogeneity of real effects across studies was quantified using the I^2^ statistic to estimate the proportion of total variability due to between-study heterogeneity.^52^ If studies were unable to be pooled in the meta-analysis, they were synthesised narratively as per Cochrane guidelines.^53^

We explored potential sources of heterogeneity by conducting: (i) subgroup analyses based on primary RCT timepoint, types of comparator intervention, adequacy of anti-inflammatory dietary intervention and risk of bias; and (ii) meta-regression (for continuous variables of: mean age, mean body mass index, proportion of female participants, difference in weight change between groups). The influence of individual studies on the overall effect size estimate was explored with a leave-one-out analysis. Small study effect was investigated by visual inspection of the funnel plots and accompanying Egger test.^54^ Analyses were performed using Stata SE V.18 (StataCorp) and RStudio (v4.2.2).^55^

The overall certainty of evidence was evaluated independently by two authors (LL, JJH) using the Grading of Recommendations Assessment, Development and Evaluation (GRADE) approach^56^ and conflicts resolved by a third author (AGC). As all included studies were RCTs, the judgement of the certainty of the evidence started as high. However, this rating was downgraded to moderate, low or very low, based on predetermined criteria: risk of bias, consistency of the reported results, indirectness of evidence, imprecision and publication bias in line with the GRADE handbook recommendations.^56^

### Protocol deviations

In our protocol, we planned to include peer-reviewed English, Norwegian, Swedish, Danish, Italian, Chinese and Cantonese articles. We modified our approach to include only RCTs in English or already translated into English, due to resource constraints (type of deviation: omission).

### Patient and public involvement

Patients and the public were not directly involved in the conduct of this systematic review. However, the review was informed by findings from our pilot trial of an anti-inflammatory dietary intervention in individuals with knee osteoarthritis.^57^ Semi-structured interviews with trial participants (n=14) highlighted HRQOL as a key concern for patients, guiding our decision to systematically evaluate HRQOL outcomes in this review.

## Results

23 RCTs were included in this systematic review (online supplemental eFigure 1). We contacted the authors of ten studies for additional data^58^,^67^ and received necessary data from five studies.^6062^,^64^ Data from four non-responding studies were imputed following Cochrane Handbook recommendations.^37^ Data from one RCT^67^ was not able to be included in the meta-analysis due to insufficient data and was summarised narratively. Only four studies reported markers of inflammation.^58 63 66 68^

### Study characteristics

HRQOL outcomes were reported for a total of 3294 participants (62% female) with a weighted mean age of 55 years (table 1 and online supplemental eTable 4). The most common chronic diseases evaluated were type two diabetes (n=8), musculoskeletal conditions (n=5) and cardiovascular diseases (n=3). All but two trials had a follow-up length of ≤1 year.^59^,^69^ 14 trials (61%) based their dietary intervention on the Mediterranean diet, seven (30%) on low and very-low carbohydrate diets, one on the DASH (4%) and one on a low-sugar, low-yeast diet (4%) (table 1). Comparator groups included usual care, medication, social support groups and healthy eating advice according to National Guidelines. Diet-only interventions were used in 16 trials (69%), multicomponent interventions were used in six (26%) and a diet and exercise combination intervention was used in one study (4%). Full details of the anti-inflammatory and CG interventions are shown in online supplemental eTable 4. Details of intervention intensity reported varied considerably, incorporating frequency and total number of contacts, total contact time, duration of the intervention and the type/number of behaviour change techniques used (online supplemental eTable 4). Sixteen (73%) trials evaluated HRQOL with one of four MOS health surveys: SF-36,^5859 62 65^,^67 70^ RAND-36,^64^ SF-20^77^ and SF-12^63 78^ (table 1); two trials used the AQoL-8D^61 79^; and single trials used the EQ-5D,^80^ CDC Healthy Days Measure^60^ and WHOQOL.^81^

**Table 1:** Summary of included trial characteristics

Study	Location	Population*	Primary chronic disease	Control type	Age, years Mean±SD	Female, n (%)	HRQOL tool/subscale†	Intervention type
Bayes 2022^81^	Australia	Males aged 18–25 years	Depression	Befriending support sessions	IG=22 ± 3 CG=23 ± 3	0 (0)	WHOQOL	MedDiet
Dolatkhah 2022^67^	Germany	Overweight/obese women aged ≥40 years	Knee osteoarthritis	Low calorie diet	IG=53 ± 7 CG=55 ± 8	60 (100)	SF-36	Anti-inflammatory diet (plus low-calorie diet)
Durrer 2021^77^	Canada	Consumers aged 30–75 years	T2DM	Usual care (guideline-based)	IG=58 ± 11 CG=59 ± 8	IG=55 (56) CG=51 (57)	SF-20 (role functioning subscale)	Carbohydrate- and energy-restricted diet
Field 2022^63^	Australia	Adults aged ≥18 years	Chronic MSK pain	Whole-food diet	IG=54 ± 15 CG=50 ± 12	IG=12 (80) CG=8 (89)	SF-12	Ketogenic diet
García-Morales, 2020^58^	Mexico	Women aged >18 years	RA	General nutritional recommendations	IG 1=51 ± 12 IG 2=46 ± 13‡ IG 3=50 ± 11 CG=49 ± 12	144 (100)	SF-36	MedDiet and exercise programme MedDiet‡ Exercise programme
Ghaseminasab-Parizi 2021^68^	Iran	Adults aged 18–70 years	RA	Usual diet+wheat	IG 1=49 ± 13‡ IG 2=55 ± 9 CG=49 ± 11	IG 1=29 (91) IG 2=33 (94) CG=32 (91)	SF-36	Anti-inflammatory diet (ie, MedDiet) + flaxseed‡ Usual diet+flaxseed
Guldbrand 2014^59^	Sweden	Adults	T2DM	Low fat diet	IG=61 ± 10 CG=63 ± 11	IG=16 (53) CG=18 (58)	SF-36	Low-carbohydrate diet
Hobday 2008^70^	United Kingdom	Adults	Chronic fatigue syndrome	Guideline-based diet	IG=44 ± 10 CG=42 ± 12	IG=22 (88) CG=21 (78)	SF-36 (role physical subscale)	Low-sugar low-yeast
Jensen 2022^62^	Denmark	Adults	T2DM, obesity	Guideline-based diet advice	IG=66 ± 7 CG=67 ± 9	IG=14 (41) CG=18 (55)	SF-36	Low-carbohydrate high-protein diet
Lundanes 2024^114^	Norway	Female adults aged 18–75 years with BMI 30–45 kg/m^2^	Lipedema	Low-energy diet	IG=48 ± 9 CG=46 ± 13	61 (100)	RAND-36	Low-carbohydrate low energy diet
Marcos-Forniol 2018^65^	Spain	Adults aged ≥70 years admitted to a single Cardiology Unit	Acute coronary syndrome	Usual care by physician	IG=76 (74–79§^)^ CG=75 (74–79§)	IG=17 (32) CG=25 (48)	SF-36	MedDiet
Masa-Font 2015^71^	Spain	Adults aged 18–65 years	Schizophrenic, schizoaffective, bipolar disorder	Usual care by physician	IG=46 ± 9 CG=47 ± 10	IG=76 (45) CG=74 (45)	SF-36	MedDiet
Michalsen 2005^72^	Germany	Adults with diagnosis of coronary artery disease	Coronary artery disease	Written advice on healthy eating	IG=59 ± 9 CG=60 ± 9	IG=10 (21) CG=13 (25)	SF-36	MedDiet
Parletta 2016^79^	Australia	Adults aged 18–65 years with diagnosed or self-reported depression forb≥2 months	Depression	Social group with food and activities	IG=44 ± 13 CG=45 ± 13	IG=54 (72) CG=51 (66)	AQoL-8d (physical health subscale)	MedDiet
Properzi 2018^61^	Australia	Adults with HS >5.5% and mean alcohol consumption <20–30 g/day	Non-alcoholic fatty liver disease	Low-fat diet	IG=51 ± 13 CG=53 ± 9	IG=11(42) CG=14 (56)	AQoL-8d	MedDiet (based on traditional Cretan diets)
Represas-Carrera, 2021^80^	Spain	Adults aged 45–75 years with ≥2 unhealthy life habits	T2DM	Guideline-based healthcare	IG=60 (range 54–67) CG=60 (range 54–66)	IG=128 (38) CG=134 (38)	EQ-5D	MedDiet
Rock 2014^66^	USA	Adults with BMI 25–45 kg/m^2^	T2DM	Guideline-based healthcare	IG1=55 ± 9 IG2=57 ± 9‡ UC=57 ± 9	IG1=35 (47) IG2=37 (48) UC=44 (58)	SF-36	Low fat Low-carbohydrate‡
Saslow 2017^73^	USA	Adults with BMI ≥25 kg/m^2^ and high HbA1c (6.5%–9%)	T2DM	Low-fat diet	IG=53 ± 10 CG=58 ± 7	IG=6 (50) CG=9 (69)	SF-36 (vitality subscale only)	Ketogenic diet
Silva 2022^74^	Portugal	Females aged 18–75 years	Fibromyalgia	General healthy eating advice	IG=60 ± 6 CG=56 ± 8	46 (100)	SF-36	Anti-inflammatory diet and low-FODMAP diet
Skӧldstam 2003^75^	Sweden	Adults starting outpatient rehabilitation at a single hospital	RA	Usual diet	IG=58 (range 33–73) CG=59 (range 35–75)	IG=21 (81) CG=20 (80)	SF-36 (role physical subscale)	MedDiet
Toobert 2003^78^	USA	Postmenopausal women aged <75 years	T2DM	Usual care	IG=61 CG=62¶	279 (100)	SF-12	MedDiet
Toobert 2011^60^	USA	Women aged 30–75 years of self-identified Latina ethnicity	T2DM	Usual care	IG=56 ± 10 CG=59 ± 10	280 (100)	CDC Healthy Days measure	MedDiet
Young 2010^76^	USA	Adults aged ≥25 years with pre-hypertension or stage one hypertension	Hypertension	General healthy eating advice	IG 1=50 ± 9‡ IG 2=50 ± 9‡ CG=50 ± 9	IG 1=174 (65)‡ IG 2=153 (57)‡ CG=172 (63)	SF-36	Established intervention‡ DASH+Established intervention

* Participant demographic data, including age, were obtained from each study’s eligibility criteria.

† Overall physical component scores and mental component scores were unable to be calculated due to missing subscale data. The next appropriate subscale was used instead.

‡ Anti-inflammatory dietary intervention group(s) used in the meta-analysis.

§ Reported in median (95%CI).

¶ From another source: the Reach and Adoption report.^115^

AQoL-8D, Assessment of Quality of Life 8-dimension instrument; BMI, body mass index; CDC, Centres for Disease Control; CG, control group; DASH, Dietary Approaches to Stop Hypertension; IG, intervention group; MedDiet, Mediterranean Diet; MSK, musculoskeletal; NR, not reported; RA, rheumatoid arthritis; SF-12, 2-Item Short Form Health Survey; SF-20, 20-Item Short Form Health Survey; SF-36, Medical Outcomes Study Short Form 36; T2DM, type two diabetes mellitus; WHOQOL, WHO Quality of Life.

### Risk of bias and quality of dietary interventions

Overall, 19 trials (83%) were judged to have ‘some concerns’, and four trials (17%) as having high risk of bias (online supplemental eFigure 2).^82^ Domain 2 (bias because of assignment to intervention) and domain 4 (measurement of the outcome) were common sources of bias in 18 (78%) and 21 (91%) trials, respectively. Missing HRQOL data was a limitation for 13 trials (57%). Only four (17%) trials were considered to have dietary interventions that were of adequate anti-inflammatory quality (online supplemental eTable 1). Six trials (26%) provided insufficient information about intervention components for an assessment of anti-inflammatory quality to be made.

### HRQOL: PCS

**Figure 1:**
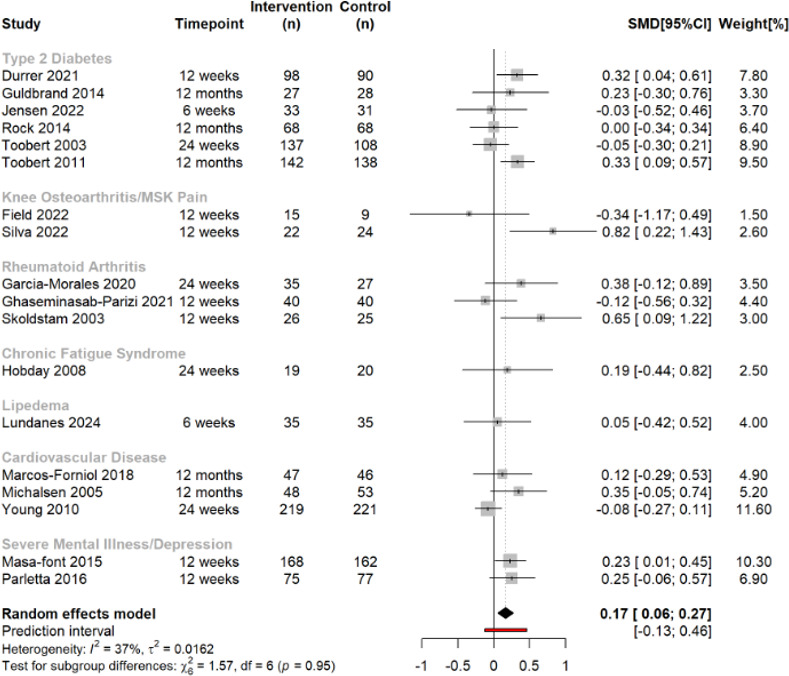
Forest plot describing the effect of anti-inflammatory dietary interventions on physical component health-related quality of life score across multiple chronic diseases. Positive SMD favours the anti-inflammatory diet group. SMD, standardised mean difference; n, number of participants; MSK, musculoskeletal.

Data pooled from 18 trials (n=2456) highlighted a small beneficial effect of anti-inflammatory dietary interventions over CGs on physical component scores of HRQOL (SMD 0.17, 95% CI 0.06 to 0.27, *I*^2^=37%) (figure 1). No disease-specific subgroup appeared to impact the overall effect size differently. Subgroup analyses suggested that physical HRQOL improvements were present at 12 weeks and 12 months (SMD 0.26, 95% CI 0.13 to 0.39, and SMD 0.22, 95% CI 0.07 to 0.38, respectively) but not at 6 weeks and 6 months (online supplemental eTable 3). Studies at high risk of bias (SMD 0.35, 95% CI 0.16 to 0.55) had a larger effect size than studies with some concerns (SMD 0.12, 95% CI 0.01 to 0.24), but neither anti-inflammatory diet quality, type of IG or CG comparison of included studies significantly influenced the overall pooled effect (online supplemental eTable 3). Meta-regression analyses revealed that the effect of anti-inflammatory diets was not associated with the proportion of women included (β 0.0001, 95% CI −0.004 to 0.004, p=0.963), mean age (β 0.002, 95% CI −0.012 to 0.016, p=0.798), mean body mass index (BMI) (β −0.021, 95% CI −0.056 to 0.015, p=0.263) or mean between-group weight change (β 0.009, 95% CI −0.029 to 0.048, p=0.633) (online supplemental eFigure 8). We found no evidence of publication bias (online supplemental eFigure 3), and findings were robust in leave-one-out analysis (online supplemental eFigure 5). The one study that could not be included in meta-analysis^67^ had some concerns regarding risk of bias and found that an anti-inflammatory diet (rated as adequate quality) resulted in no greater improvement in physical component HRQOL compared with a low-calorie diet.

### HRQOL: MCS

**Figure 2:**
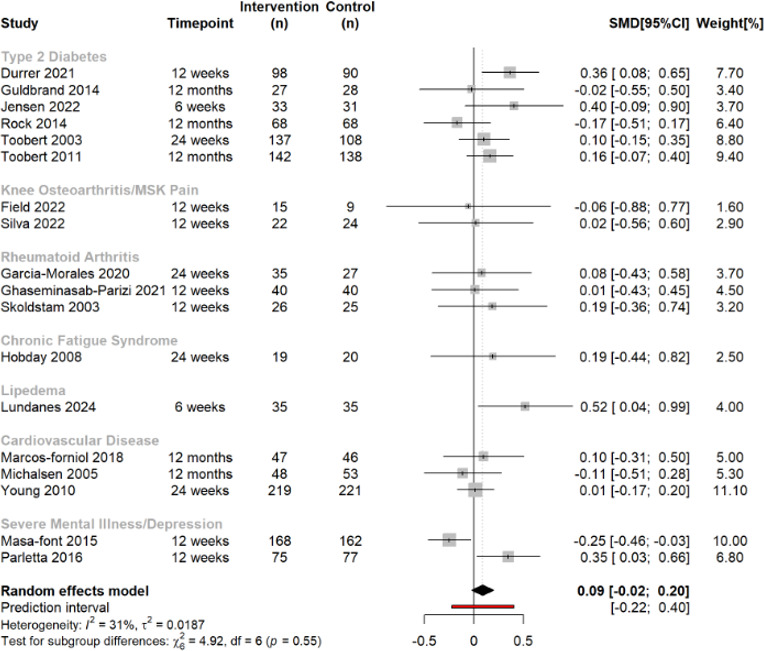
Forest plot describing the effect of anti-inflammatory dietary interventions on mental component health-related quality of life score across multiple chronic diseases. Positive SMD favours the anti-inflammatory diet group. MSK, musculoskeletal; REML, random effects meta-analysis; SMD, standardised mean difference.

Summary estimates from 18 trials (n=2456) indicated that anti-inflammatory dietary interventions were not associated with change in MCS compared with CGs (SMD 0.09, 95% CI −0.02 to 0.20, *I*^2^=31%) (figure 2). No disease-specific subgroup appeared to impact the overall effect size differently. Subgroup analyses demonstrated that, compared with multicomponent interventions, diet-only interventions lead to greater improvements in MCS (SMD 0.14, 95% CI 0.04 to 0.24) (online supplemental eTable 2). Heterogeneity was not explained by any other study-level factor, except type of intervention. Meta-regression analyses revealed that the effect of anti-inflammatory diets was not associated with the proportion of women included (β 0.003, 95% CI −0.001 to 0.007, p=0.147), mean age (β 0.002, 95% CI −0.012 to 0.017, p=0.769), mean BMI (β 0.018, 95% CI −0.017 to 0.053, p=0.309) or mean between-group weight change (β 0.015, 95% CI −0.027 to 0.057, p=0.481) (online supplemental eFigure 9). We found no evidence of publication bias (online supplemental eFigure 4), and findings were robust in leave-one-out analysis (online supplemental eFigure 6). The one study that could not be included in the meta-analysis^67^ had some concerns regarding risk of bias, but its results were consistent with the pooled effects from the meta-analysis.

### HRQOL: General score

**Figure 3:**
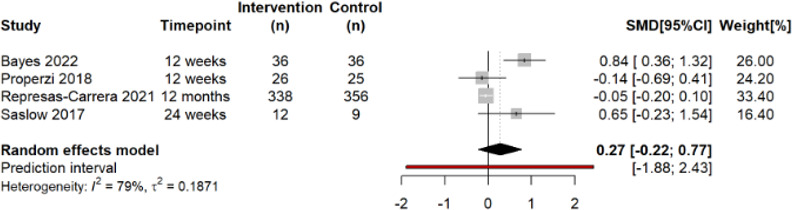
Forest plot describing the effect of anti-inflammatory dietary interventions on general component score across chronic disease subgroups. Positive SMD favours the anti-inflammatory diet group. REML, random effects meta-analysis; SMD, standardised mean difference.

Anti-inflammatory dietary interventions were not associated with changes in general HRQOL score compared with CGs. Summary estimates based on four trials (n=838) indicated a moderate non-statistically significant effect on general HRQOL score (SMD 0.27; 95% CI −0.22 to 0.77, *I*^2^=79%) (figure 3). Assessment for publication bias and further subgroup and meta-regression analyses were not possible due to the limited number of studies. Findings were robust in leave-one-out analyses (online supplemental eFigure 7).

### Overall certainty of evidence

Certainty of evidence in accordance with GRADE was low for PCS and MCS and very low for the general HRQOL score. The certainty of evidence was downgraded for most outcomes due to indirectness, as the dietary interventions varied in composition across studies (online supplemental eTable 3).

## Discussion

### Main findings

This systematic review and meta-analysis of 23 trials with HRQOL data from 3294 adults with at least one chronic disease demonstrated that anti-inflammatory diets may lead to small, but possibly not clinically relevant improvements in physical HRQOL. We found no evidence of an effect that anti-inflammatory diets affected mental component or general HRQOL, but the certainty of evidence was low to very low, and the quality of anti-inflammatory diets was poor in most trials.

Our review is the first to evaluate the effect of anti-inflammatory diets on HRQOL by combining data from adults with different index chronic diseases and comorbidities. The results suggest that anti-inflammatory diets may improve physical HRQOL in individuals with chronic disease, but, importantly, do not negatively affect any aspect of HRQOL. Our stratified analyses (online supplemental eTable 2) and meta-regressions (online supplemental eFigures 8 and 9) indicate that the effects on physical component HRQOL were unaffected by age, sex, baseline BMI, length of follow-up and weight change during the dietary intervention. The benefit in physical HRQOL observed is consistent with existing observational data indicating that higher adherence to an anti-inflammatory (Mediterranean) diet is associated with a statistically significant reduction in overall mortality.^83^ Our results build on disease-specific systematic reviews of RCTs that show that anti-inflammatory dietary interventions improve disease-specific clinical markers across a variety of chronic conditions. For example, anti-inflammatory diets increase rates of diabetes remission (defined as HbA1c<6.5%),^84^ reduce cardiovascular disease risk^85^ and significantly improve pain in rheumatoid arthritis compared with usual diets.^25^ The benefits we observed for physical HRQOL are likely to be somewhat related to the benefits observed on disease-specific markers and may also be related to other aspects of health and general wellness, including improved sleep,^86^ sustained satiety^87^ and self-reported and functional pain.^15 88^ It is important to note that the effects of an anti-inflammatory diet on physical HRQOL we observed were of low certainty evidence. The effect size we observed may change with further high-quality RCTs.

Anti-inflammatory diets were not associated with statistically significant effects in mental component or general HRQOL, although these results were of low and very low certainty evidence, respectively. It is unclear why the effects of anti-inflammatory dietary interventions were attenuated for MCS compared with PCS. In trials reporting a general HRQOL outcome, the point estimate (SMD 0.27) reflected a small effect size and was accompanied by large 95% CIs, due to the lack of trials (n=4), mostly with small participant numbers.

The pooled effect estimates we obtained across all components of HRQOL may underestimate the true effect of anti-inflammatory diets due to the nature of the original RCT interventions, many of which were not truly anti-inflammatory. For example, some trials modified essential components in their dietary intervention (eg, substituting extra virgin olive oil with seed oils or margarine) to suit their study populations.^58 70^ While anti-inflammatory diets can (and should) be individualised to an extent to support adherence, data from ecologic comparisons and systematic reviews strongly suggest that key components and an overall nutrient profile must be preserved to maximise the possible health benefits.^89^,^91^ Overall, 18 of 23 trials in our review evaluated a poor-quality anti-inflammatory dietary intervention. These findings align with previous research, which observed that modified anti-inflammatory diets—without core health-promoting elements and nutritional compositions—result in diluted health benefits observed in typical anti-inflammatory diets.^89 92^ The lack of a uniformly accepted definition of anti-inflammatory diet may also contribute to the variability in our results.

The relatively small effect of anti-inflammatory diets observed could be due to the characteristics of chronic diseases in participants. We *a priori* decided to combine all trials of participants with at least one chronic disease, irrespective of the index/primary condition. The effect sizes of trials for each specific condition appeared to be similarly heterogeneous, and no single condition appeared to considerably impact the overall pooled effect. This is consistent with systemic inflammation being involved in a wide variety of mental and physical chronic diseases that account for 50% of all deaths globally.^93^ However, over half of the studies recruited participants based on having only mild disease severity. It is likely that these participants (with mild disease) report less impaired HRQOL scores at trial enrolment and are therefore less likely to improve because of an anti-inflammatory intervention compared with those with long-standing diseases with more impaired baseline values.^94 95^ This aligns with findings from a recent meta-analysis investigating treatment effects on HRQOL in 19 chronic diseases, which highlighted associations between larger effect sizes and lower pre-treatment values, reflecting later disease stages.^96^ Despite multimorbidity increasing with age,^97^ our meta-regression analyses showed that mean age of the included trials did not explain heterogeneity in the effect on HRQOL outcomes.

We observed significant heterogeneity among the types of dietary interventions and key components of the included trials, which is a commonly reported limitation in meta-analyses on this topic.^98^ Notably, only six (26%) of the included trials had a primary outcome of and were appropriately powered for, HRQOL. Anti-inflammatory dietary advice was delivered in multicomponent programmes in three studies reporting positive associations with physical HRQOL,^65 71 72^ but we could not isolate the independent influence of diet on the observed outcomes. Previous research emphasises that multicomponent interventions promoting strength and mobility, cognitive training^99^ and social support^100 101^ are more effective for improving HRQOL than single interventions for maintaining good health in older adults. Regular contact and social support (eg, with researchers, clinicians, family, peers) is also associated with greater effectiveness and adherence in studies of dietary interventions.^102 103^ However, the frequency of contact in included interventions varied widely, and due to the inconsistent reporting of intervention adherence and dietary intake, we were not able to assess the effect of adherence on the HRQOL outcomes we observed.

Similar to previous systematic reviews of HRQOL in other conditions,^104^,^106^ we were able to assess the effect of anti-inflammatory diets on physical and mental components of HRQOL separately due to the majority of trials (n=14, 61%) measuring HRQOL with the SF-36 instrument (or similar versions).^39^ However, while the SF-36 is valid and reliable,^107 108^ previous studies suggest that the responsiveness of the physical and mental component scores may differ depending on disease characteristics and nature of the interventions used.^109 110^ The low and very low certainty evidence associated with our findings highlights the need for future well-designed RCTs to confirm the effect of anti-inflammatory diets on HRQOL. Our review can help underpin the planning and methodological conduct (eg, sample size calculations) for future dietary trials for chronic disease and in particular, multimorbidity, which is recognised as a key priority in global health research.^111 112^

### Limitations

Several limitations should be considered when interpreting our findings. First, there was substantial heterogeneity between studies in terms of IG and CG treatments and intensity, types of dietary interventions and methods used to assess adherence and HRQOL. We accounted for these in our GRADE approach, and while the quality of trials was generally low, our sensitivity analysis showed that very few individual studies significantly influenced the results of the meta-analyses. Moreover, subgroup analyses indicated that differences in comparator type did not significantly alter the direction or magnitude of the observed effects. There was no clear evidence of any associations between setting, age, delivery mode of the target group and effectiveness. We were unable to account for the potential influence of specific interventions delivered in the CGs; however, our subgroup analysis did show that the pooled effects did not differ between usual care CGs and active intervention CGs. We were also unable to determine the potential influence of the presence of more than one chronic disease due to the rates of multimorbidity being poorly reported in the original trials. Additionally, although the current review aimed to capture a variety of dietary patterns, the results show that the research has been dominated by studies on the most popular types of diets - specifically, the Mediterranean diet. Our interpretation was hampered by poor descriptions of interventions, limited reporting of assessment and adherence data and intervention fidelity, and the observation that only five of 23 studies met criteria for ‘adequate’ anti-inflammatory dietary quality. Few studies assessed and reported the anti-inflammatory potential of their dietary interventions, making it challenging to determine whether variations in dietary adherence, nutrient composition or anti-inflammatory potential influenced the outcomes. Blinding of participants in most included studies was not possible, a well-established limitation in dietary research with free-living participants.^113^ In addition, most studies relied on self-reported dietary intake data without biomarker validation, which may have resulted in an overreporting of dietary adherence and underestimation of anti-inflammatory diet effect on HRQOL. We planned to explore the potential mediating role of systemic inflammation (eg, molecular biomarkers related to low-grade inflammation, such as interleukin-6, tumour necrosis factor and C-reactive protein) and physical activity changes. Lack of data in the included studies precluded this. Finally, we cannot comment on the effectiveness of the provider of the interventions. Although dietary interventions alone were more effective than multicomponent interventions for mental HRQOL, but similarly effective for physical HRQOL, whether effectiveness was affected by delivery personnel (ie, dietitian vs non-dietitian) was unclear.

## Conclusion

In this systematic review of adults with at least one chronic disease, anti-inflammatory diets led to small and possibly not clinically relevant, improvements in physical component HRQOL. Anti-inflammatory diets did not impact mental component or general HRQOL. The findings must be considered in the context of the limitations in the primary studies as the certainty of evidence was low to very low.

## Supplementary material

10.1136/bmjnph-2025-001257 online supplemental file 1

## Data Availability

Data are available upon reasonable request.
