# Supplementary material for: Effect of anti-inflammatory diets on health-related quality of life in adults with chronic disease: a systematic review and meta-analysis
Source: BMJ Nutr Prev Health. 2025 Jun 10;8(1):e001257. doi: 10.1136/bmjnph-2025-001257 (PMC12322563; doi:10.1136/bmjnph-2025-001257)
Supplement: online supplemental file 1 [file bmjnph-8-1-s001.pdf]

Effect of anti-inflammatory diets on health-related quality of life in adults with chronic disease: a systematic review and meta-analysis

Table of Contents

eMethods 1. Systematic search strategy

eMethods 2. Description of how standardised mean differences and its variance were acquired

eTable 1. Assessment of anti-inflammatory dietary quality

eTable 2. Meta-analyses stratified by study-level characteristics

eTable 3. GRADE Assessment

eTable 4. Anti-inflammatory diet and control group interventions

eFigure 1. PRISMA flowchart

eFigure 2. Risk of bias summary graph

eFigure 3. Publication bias (physical component score)

eFigure 4. Publication bias (mental component score)

eFigure 5. Leave-one-out analyses (physical component score)

eFigure 6. Leave-one-out analyses (mental component score)

eFigure 7. Leave-one-out analyses (general HRQOL)

eFigure 8. Meta-regressions examining the relationship between physical component score and study-level characteristics

eFigure 9. Meta-regressions examining the relationship between mental component score and study-level characteristics

References

eMethods 1. Systematic search strategy

**MEDLINE**	(keto* OR atkins OR anti-inflam* OR low-inflam* OR Mediterranean OR low-carb* OR nordic OR okinawan OR carbohydrate-restrict* OR MIND or DASH OR paleo* OR carnivor* OR lectin-free) adj2 (food* OR program OR regim* OR diet*).mp. OR Diet, Ketogenic/ or Diet, Carbohydrate-Restricted/ OR Diet, Mediterranean/ OR Diet, High-Fat/ OR Diet, High-Protein Low Carbohydrate/ OR Diet, Paleolithic<br>**AND**<br>Quality of life/ OR "quality of life".mp OR (HrQoL OR HR-QOL OR SF-12 OR EQ-5D OR EQ5D OR Euroqol OR SF-36 OR SF-20 OR SF-8 OR WHOQOL-100 OR AQoL OR CASP-16 OR EQ-VAS OR PROMIS OR EUROHIS-QoL).mp.<br>**AND**<br>Clinical Trials as Topic/ OR Randomized controlled trials as Topic/ OR Control Groups/ OR Random Allocation/ OR Clinical Trial/ OR (Randomi* OR "Clinical trial" OR "control group").mp
**Embase**	(keto* OR atkins OR anti-inflam* OR low-inflam* OR Mediterranean OR low-carb* OR nordic OR okinawan OR carbohydrate-restrict* OR MIND or DASH OR paleo* OR carnivor* OR lectin-free) adj2 (food* OR program OR regim* OR diet*).mp. OR Ketogenesis/ OR ketogenic diet/ OR Mediterranean diet/ OR Low carbohydrate diet/ OR Paleolithic diet/ OR DASH diet/ OR MIND diet/ OR Modified Atkins diet/ OR Nordic diet/ OR Okinawan diet/ OR Atkins diet/<br>**AND**<br>[mh “Quality of Life”] OR "quality of life".mp. OR (HrQoL OR HR-QOL OR SF-12 OR EQ-5D OR EQ5D OR Euroqol OR SF-36 OR SF-20 OR SF-8 OR WHOQOL-100 OR AQoL OR CASP-16 OR EQ-VAS OR PROMIS OR EUROHIS-QoL).mp.<br>**AND**<br>Clinical Trial/ OR Randomized Controlled Trial/ OR Controlled Study/ OR Single-Blind Procedure/ OR Double-Blind Procedure/ OR Randomization/ OR Controlled clinical trial/ OR (randomi* OR control group OR "clinical trial").mp.
**CINAHL**	TX ((keto* OR atkins* OR anti-inflam* OR low-inflam* OR Mediterranean OR low-carb* OR nordic OR okinawan OR carbohydrate OR MIND OR DASH OR paleo* OR carnivor* OR lectin-free) N2 (food* OR program OR regim* OR diet* OR restrict*)) OR (MH "Diet, Paleolithic") OR (MH "Diet, Low Carbohydrate") OR (MH "Diet, Nordic") OR (MH "Diet, Ketogenic") OR (MH "Diet, High Protein") OR (MH "Mediterranean Diet") OR (MH "DASH Diet")<br>**AND**<br>(MH "Quality of Life+") OR (MH "Quality-Adjusted Life Years") OR TX (HrQoL OR HR-QOL OR HRQL OR SF-12 OR EQ-5D OR EQ5D OR Euroqol OR SF-36 OR SF-20 OR SF-8 OR WHOQOL-100 OR AQoL OR CASP-16 OR EQ-VAS OR PROMIS OR EUROHIS-QoL) OR TX (quality-of-life)<br>**AND**<br>(MH "Randomized Controlled Trials") OR (MH "Clinical Trials") OR (MH “Control Group”) OR TX (randomi* OR "control group" OR "clinical trial")
**Web of Science**	((keto* OR atkins OR anti-inflam* OR low-inflam* OR Mediterranean OR low-carb* OR nordic OR okinawan OR carbohydrate-restrict* OR MIND or DASH OR paleo OR carnivor* OR lectin-free) near/2 (food* OR program OR regim* OR diet* OR restrict*))<br>**AND**<br>("quality of life")<br>**AND**

	(HrQoL OR HR-QOL OR SF-12 OR EQ-5D OR EQ5D OR Euroqol OR SF-36 OR SF-20 OR SF-8 OR WHOQOL-100 OR AQoL OR CASP-16 OR EQ-VAS OR PROMIS OR EUROHIS-QoL)<br>**AND**<br>("control group" OR "Clinical trial" OR randomi*)
**CENTRAL**	(keto* OR atkins* OR anti-inflam* OR low-inflam* OR Mediterranean OR low-carb* OR nordic OR okinawan OR carbohydrate OR MIND OR DASH OR paleo* OR carnivor* OR lectin-free) NEAR/2 (food* OR program OR regim* OR diet* OR restrict*) OR [mh "Diet, Paleolithic"] OR [mh "Diet, Carbohydrate-Restricted"] OR [mh "Diet, High Protein Low-Carbohydrate"] OR [mh "Mediterranean Diet"] OR [mh “Dietary Approaches to Stop Hypertension”]<br>**AND**<br>“quality of life” OR (HrQoL OR HR-QOL OR SF-12 OR EQ-5D OR EQ5D OR Euroqol OR SF-36 OR SF-20 OR SF-8 OR WHOQOL-100 OR AQoL OR CASP-16 OR EQ-VAS OR PROMIS OR EUROHIS-QoL)<br>**AND**<br>[mh “Clinical Trial”] OR [mh “Control Groups”] OR [mh “Randomized Controlled Trial”] OR (randomi* OR control group OR "clinical trial")

eMethods 2. Description of how standardised mean differences and its variance were acquired

**Study**	**Analysis**	**Notes**
Bayes et al, 2022[1]	Complete case	Intervention and control group means and SDs for the change between follow-up and baseline were available to calculate the SMD and its variance.
Dolatkah et al, 2022[2]	Intention to treat	Sufficient information was not available to calculate SMD and its variance, hence study was not included in the quantitative meta-analysis.
Durrer et al, 2021[3]	Intention to treat	The mean and 95% CI for the between-group change was available. This was used to calculate the SMD and its variance assuming equal SDs between the groups.
Field et al, 2022[4]	Complete case	Intervention and control group means and SDs for the change between follow-up and baseline were available to calculate the SMD and its variance.
García-Morales et al, 2020[5]	Complete case	Intervention and control group means and SDs for the change between follow-up and baseline were available to calculate the SMD and its variance.
Ghaseminasab-Parizi et al, 202[6]	Intention-to-treat and complete case	Intervention and control group means and SDs for the change between follow-up and baseline were available to calculate the SMD and its variance.
Guldbrand et al, 2014[7]	Complete case	Intervention and control group means and SDs at the follow-up and baseline were available. Correlation between baseline and follow-up calculated from Michalsen et al.[8] was used to calculate SMD and its variance for the between-group change.
Hobday et al, 2008[9]	Complete case	Intervention and control group means and SDs at the follow-up and baseline were available. Correlation between baseline and follow-up calculated from Michalsen et al.[8] was used to calculate SMD and its variance for the between-group change.
Jensen et al, 2022[10]	Complete case	Intervention and control group means and SDs for the change between follow-up and baseline were available to calculate the SMD and its variance.
Lundanes et al, 2024[11]	Intention-to-treat	Intervention and control group means and SDs for the change between follow-up and baseline were available to calculate the SMD and its variance.

Marcos-forniol et al, 2018[12]	Complete case	Intervention and control group median (IQR) for the change between follow-up and baseline were used to estimate means and SDs. Assuming a large enough sample size and normal distribution of outcome, we approximated the means using medians and IQR as approximately 1.35 SDs. These were subsequently used to calculate standardised mean difference and its variance for the between-group change.
Masa-Font et al, 2015[13]	Intention-to-treat	Intervention and control group means and 95% CIs for the change between follow-up and baseline were available. CIs were used to obtain estimated SDs for the intervention and control groups, and these were then used to calculate the SMD and its variance for the between-group change.
Michalsen et al, 2005[8]	Complete case	Intervention and control group means and SDs for the change between follow-up and baseline were available to calculate the SMD and its variance.
Parletta et al, 2016[14]	Intention-to-treat	The mean and 95% CI for the between-group change was available. This was used to calculate SMD and its variance assuming equal SDs between the groups.
Properzi et al, 2018[15]	Intention-to-treat	Intervention and control group means and SDs at follow-up and baseline were available. Correlation between baseline and follow-up calculated from Michalsen et al.[8] was used to additionally calculate SMD and its variance for the between-group change.
Represas-Carrera et al, 2021[16]	Intention-to-treat	The mean and 95% CI for the change between follow-up and baseline groups were available. This was used to calculate SMD and its variance of the between-group change assuming equal SDs between the groups.
Rock et al, 2014[17]	Complete case	Intervention and control group means and SDs at the follow-up and baseline were available. Correlation between the baseline and follow-up calculated from Michalsen et al.[8] was used to calculate SMD and its variance for the between-group change.
Saslow et al, 2017[18]	Complete case	The mean and 95% CI for the change between follow-up and baseline groups were available. This was used to calculate SMD and its variance assuming equal SDs between the groups.
Silva et al, 2022[19]	Complete case	Intervention and control group means and SDs for the change between follow-up and baseline were available to calculate the SMD and its variance.

Sköldstam et al, 2003[20]	Complete case	Intervention and control group means and SDs for the change between follow-up and baseline were available to calculate the SMD and its variance for the between-group change.
Toobert et al, 2003[21]	Complete case	Intervention and control group means and SDs at the follow-up and baseline were available. Correlation between the baseline and follow-up calculated from Michalsen et al.[8] was used to calculate SMD and its variance for the between-group change.
Toobert et al, 2011[22]	Intention-to-treat and complete case	Intervention and control group means and SDs at the follow-up and baseline were available. Correlation between the baseline and follow-up calculated from Michalsen et al.[8] was also used to calculate SMD and its variance for the between-group change.
Young et al, 2010[23]	Complete case	Intervention and control group means and standard errors for the change between follow-up and baseline were available. These were used to calculate the SMD and its variance for the between-group change.

MD, mean difference; SD, standard deviation; SMD, standardised mean difference; CI, confidence interval

The data required for meta-analysis was the SMD (and its variance) for the difference between the intervention and control groups change score.

When only follow-up data were available for studies using the SF-36 outcome (not the change from baseline data), we estimated the SMD and its variance using a correlation coefficient of 0.5 (calculated from Michaelsen et al.[8]).

eTable 1. Assessment of anti-inflammatory dietary quality

Study	Criterion 1	Criterion 2	Criterion 3	Overall
Bayes 2022[81]	ii	ii	NR	Adequate
Dolatkhah 2022[66]	ii	ii	iii	Adequate
Durrer 2021[77]	NR	NR	iii	Unable to determine
Field 2022[62]	ii	i	iii	Inadequate
García-Morales, 2020[57]	NR	ii	i	Inadequate
Ghaseminasab-Parizi 2021[68]	iii	iii	ii	Adequate
Guldbrand 2014[58]	NR	NR	iii	Unable to determine
Hobday 2008[70]	i	ii	NR	Inadequate
Jensen 2022[61]	ii	ii	ii	Inadequate
Lundanes 2024[83]	ii	ii	ii	Inadequate
Marcos-Forniol 2018[64]	NR	NR	NR	Unable to determine
Masa-Font 2015[71]	NR	NR	NR	Unable to determine
Michalsen 2005[72]	NR	NR	NR	Unable to determine
Parletta 2016[79]	NR	ii	NR	Inadequate
Properzi 2018[60]	ii	ii	ii	Inadequate
Represas-Carrera, 2021[80]	ii	ii	NR	Inadequate
Rock 2014[65]	ii	ii	NR	Inadequate
Saslow 2017[73]	i	i	iii	Inadequate
Silva 2022[74]	ii	ii	NR	Adequate
Sköldstam 2003[75]	ii	ii	NR	Inadequate
Toobert 2003[78]	ii	ii	NR	Inadequate
Toobert 2011[59]	NR	NR	NR	Unable to determine
Young 2010[76]	i	ii	NR	Inadequate

NR, not reported

Anti-inflammatory dietary quality was assessed using a predefined system developed for this review. Dietary interventions reported by included studies were rated as adequate, inadequate, or not reported (hence unable to determine), based on their potential to reduce systemic inflammation. This rating considered three criteria: Criterion1 – inclusion of anti-inflammatory foods and nutrients (i=included none, ii=included some, iii=included all; Criterion 2 – exclusion of pro-inflammatory components (i=removed none, ii=removed some, iii=removed all); and Criterion 3 – the carbohydrate content of the diet (i=carbohydrate >45% of total energy intake, ii=20-45% of total energy intake, iii=<20% of total energy intake). Two authors (LL, BD) independently applied the scoring system to all included studies, as prespecified in the review protocol. The overall rating for each study was determined based on the scoring of all three criteria, as well as taking into consideration the level of detail provided in each study and how the overall dietary intervention aligned with broad anti-inflammatory diet principles.

eTable 2. Meta-analyses stratified by study-level characteristics*

	Number of studies	SMD (95% CI)	P value
**Physical component score**			
**Primary timepoint**			0.973†
**6 weeks**	2	0.01 (-0.33, 0.35)	
**12 weeks**	7	**0.26 (0.13, 0.39)**	
**6 months**	4	-0.02 (-0.16, 0.12)	
**12 months**	5	**0.22 (0.07, 0.38)**	
**Diet quality**			0.670‡
**Unknown**	6	0.27 (0.15 to 0.40)	
**Adequate**	3	0.22 (-0.32, 0.76)	
**Inadequate**	9	0.07 (-0.08, 0.21)	
**Risk of bias**			0.08497^
**Some concerns**	14	**0.12 (0.01, 0.24)**	
**High**	4	**0.35 (0.16, 0.55)**	
**Type of intervention**			0.555
**Multicomponent**	3	**0.23 (0.06, 0.40)**	
**Diet only**	15	**0.15 (0.02, 0.28)**	
**Type of control**			0.653
**Active treatment**	10	0.14 (-0.01, 0.30)	
**Usual care**	8	**0.19 (0.05, 0.34)**	
**Mental component score**			
**Primary timepoint**			0.145†
**6 weeks**	2	**0.46 (0.12, 0.81)**	
**12 weeks**	7	0.10 (-0.13, 0.32)	
**6 months**	4	0.05 (-0.09, 0.19)	
**12 months**	5	0.02 (-0.14, 0.18)	
**Diet quality**			0.577‡
**Unknown**	6	0.04 (-0.16, 0.25)	
**Adequate**	3	0.19 (-0.15, 0.53)	
**Inadequate**	9	0.09 (-0.03, 0.21)	
**Risk of bias**			0.750^
**Some concerns**	14	0.06 (-0.06, 0.19)	
**High**	4	0.12 (-0.07, 0.32)	
**Type of intervention**			**0.021**
**Multicomponent**	3	-0.15 (-0.34, 0.05)	
**Diet only**	15	**0.14 (0.04, 0.24)**	
**Type of control**			0.226
**Active treatment**	10	0.16 (0.00, 0.31)	
**Usual care**	7	0.02 (-0.13, 0.16)	

Abbreviations: SMD, standardised mean difference; CI, confidence interval

*Bold p-values represent statistical significance ($p<0.05$). P values represent difference between stratified groups unless indicated otherwise.

† Primary timepoint modelled as continuous variable and p-value derived from meta-regression analysis.

‡ P-value for diet quality represents difference between adequate and inadequate diet quality pooled subgroups only.

eTable 3. GRADE of evidence assessment for key outcomes

Key Outcome	Study Design^	Risk of Bias	Inconsistency	Indirectness	Imprecision	Publication Bias	GRADE
Physical component score	18 RCTs n = 2457	**Serious (-1):** 4 studies with high overall risk of bias and 14 with some concerns mostly due to a lack of or incomplete blinding of outcome assessors	**Serious (-1):** Statistically significant moderate unexplained heterogeneity, I^2 = 36%, p = 0.061	**Not serious (0):** Different definitions of anti-inflammatory diets, 4 had multicomponent interventions	**(0)** Pooled SMD: 0.17 (95%CI 0.06 to 0.27) n = 2457	**(0)** Not suspected p = 0.322	LOW
Mental component score	18 RCTs n = 2456	**Serious (-1):** 4 studies with high overall risk of bias and 14 with some concerns, mostly due to a lack of or incomplete blinding of outcome assessors	**Serious (-1):** Statistically significant moderate heterogeneity partially explained only by type of intervention (multicomponent), I^2 = 31%, p = 0.101	**Not serious (0):** Different definitions of anti-inflammatory diets, 3 had multicomponent interventions	**(0)** Pooled SMD: 0.09 (95%CI -0.02 to 0.20) n = 2457	**(0)** p = 0.325	LOW
General health component score	4 RCTs n = 838	**Serious (-1):** All studies with some concerns	**Serious (-2):** Statistically significant high unexplained heterogeneity, I^2 = 81%, p = 0.001	**Not serious (0):** 1 multicomponent intervention	**Serious (-1)** Pooled SMD: 0.27 (95%CI -0.22 to 0.77) n = 838	Not tested (n<10)	VERY LOW

GRADE, Grading of Recommendations, Assessment, Development, and Evaluations; RCT, randomised controlled trial; RoB, risk of bias; SMD, standardised mean difference; CI, confidence intervals

^Grading begins at HIGH quality as all studies are RCTs.

Grade of evidence in this report is assigned using the GRADE system, which has 4 categories HIGH, MODERATE, LOW or VERY LOW. Evidence is initially assigned as HIGH if coming from a randomised trial; LOW from observational studies; VERY LOW from other evidence. The grade of evidence is then reduced if there are serious (-1) or very serious (-2) limitations to study quality or uncertainties about directness of association; important inconsistency (-1), imprecise or sparse data (-1) or a high probability of reporting bias (-1). Grade of evidence is increased if strong evidence of association is seen (e.g., RR >2 or <0.5) from ≥2 observational studies with no plausible confounders (+1) or very strong direct evidence (RR >5 or <0.2) with no major threats to validity (+2); if there is evidence of a dose-response gradient (+1) or if all plausible confounders would have reduced the effect/association seen (+1). The interpretation of GRADE evidence assessments is that for HIGH certainty evidence further research is very unlikely to change our confidence in the estimate of effect; for MODERATE certainty evidence further research is likely to have an important impact on our confidence in the estimate of effect and may change the estimate; for LOW certainty evidence further research is very likely to have an important impact on our confidence in the estimate of effect and is likely to change the estimate; and for VERY LOW certainty evidence any estimate of effect is very uncertain. Further detailed explanation of GRADE can be found at:
http://www.gradeworkinggroup.org/publications/Grading_evidence_and_recommendations_BMJ.pdf

eTable 4. Anti-inflammatory diet and control group interventions

Study	Participants (baseline)	Intervention group	Intervention frequency and duration	Control group	Control frequency and duration	Dietary assessment	Compliance	Follow-up
Bayes 2022[82]	IG = 38<br>CG = 37	Nutritionist-led MD education supported by booklet, online daily diet history, and $50 food hamper	60-minute appointments at baseline, 6 and 12 weeks	Befriending support sessions with researcher and $50 Hoyts gift card	60-minute appointments at baseline, 6 and 12 weeks	CSIRO Healthy Diet Score, adapted MEDAS, daily diet recorded on mobile widget	After 12 weeks, MEDAS scores were significantly higher in IG than control	12 weeks
Dolatkhah 2022[67]	IG = 30<br>CG = 30	Dietitian-led AI diet in addition to low calorie diet	NR	Low-calorie diet 500kcal less than individual energy requirements (C55-60%EI, P10-15%EI, F<30%EI)	NR	3-d food record	35 (65%) reported being adherent every day, 14 (26%) most days, and 5 (9%) sometimes	8 weeks
Durrer 2021[78]	IG = 98<br>CG = 90	Commercial weight loss diet supplemented with whole foods (C<50g, F ~35-45g, P110-120g, ~850-1100kcal/day)	Weekly pharmacy visits for 12 weeks	Standard advice as per 2013 Diabetes Canada Clinical Practice Guidelines	One pharmacy visit at 12 weeks	3-d food record	NR	12 weeks
Field et al 2022[63]	IG = 15<br>CG = 9	3-week run-in WFD followed by physiotherapist-led WFKD (C30-50 g/day) to achieve nutritional ketosis (ketones 0.5-3.0 mmol/L)	Fortnightly contact with physiotherapist	Continuation of WFD after run-in phase	Fortnightly contact with physiotherapist	Online 24-h food recall	At 12 weeks, 38% of IG reported continuing the diet most of the time, 50% in CG	12 weeks
García-Morales et al 2020[58]	IG 1 = 36<br>IG 2 = 40[§]<br>IG 3 = 37<br>CG 3 = 31	Dietitian-led individualised MD (C50EI%, F30EI%, P20EI%)	One visit at baseline and 24 weeks	General nutritional recommendations	No visits	24-h diet recall	NR	24 weeks
Ghaseminasab-Parizi 2021[69]	IG 1 = 40[§]<br>IG 2 = 40<br>CG = 40	Dietitian-led AI diet (heavily modified MD, C55EI%, P15EI%, F30EI%)	Weekly phone calls	Usual diet (with target C55EI%, P15EI%, F30EI%)	Weekly phone calls	3-d food record, return of unconsumed powders to monitor consumption	NR	12 weeks

Guldbrand 2014[59]	IG= 30<br>CG= 31	Dietitian-led LC diet education (F50EI%, C20EI%, P30EI%)	Group meetings at baseline, 2, 6, and 12 months	Dietitian-led traditional LFD as per Swedish guidelines (F30E%, C55-60EI%, P10-15EI%)	Group meetings at baseline, 2, 6, and 12 months	3-d food record used to measure energy intake	At 24 months, 58% in LFD and 60% in LC diet met targets	2 years
Hobday et al 2008[71]	IG = 25<br>CG = 27	Low sugar low yeast diet based on the *Beat Candida* Cookbook	3 visits and monthly phone contact	Usual care per Department of Health guidelines	3 visits and monthly phone contact	3-d food records, with diet histories taken from those who did not fill them in	6 (24%) IG and 18 (67%) CG participants were fully compliant at 24 weeks	24 weeks
Jensen et al 2022[62]	IG = 34<br>CG = 33	Fully-provided hypocaloric CRHP diet (C30%EI, P30%EI, F40%EI)	Meals were distributed biweekly for 6 weeks	Fully-provided conventional diabetes diet (C50%EI, P17%EI, F33%EI)	Meals were distributed biweekly for 6 weeks	24-h urine samples, food records collected twice weekly[1]	NR	6 weeks
Lundanes 2024[84]	IG = 35<br>CG = 35	Low-energy LC diet (1200kcal/day, C25%E, P20%E, F55%E)	Weekly phone or in-person follow-ups, including assessment of ketosis using Ketostix	Low-energy diet (1200kcal/day, C60%E, P20%E, F20%E)^	Weekly phone or in-person follow-ups, including assessment of ketosis using Ketostix	Daily food records used to measure energy (kcal/day) and macronutrients (g/day, E%) based on Norwegian Food Composition Table	IG had average intakes 1176.4 (29.6) kcal/day, C23%E, P23%E, F58%E.<br>Average CG intake was 1194.8 (109.0), C69%E, P19%E, F18%E	8 weeks
Marcos-Forniol 2018[65]	IG = 64<br>CG = 63	MDT-led secondary CHD prevention program with MD component	Baseline educational session and 3 physician appointments	Usual care	NR	9-item composite MD index calculated from FFQ	Slight improvement in MD adherence in IG at follow-up compared to baseline but worsened in CG	12 months
Masa-Font et al, 2015[72]	IG = 169<br>CG = 163	Nurse-led MD education plus PA program	16 20-minute twice weekly group sessions and	Usual care	Usual psychiatrist check-ups every 2 months	24-h food records, PREDIMED score.	49% of individuals attended ≥60% of the sessions. No significant differences in PREDIMED score.	52 weeks

			24 40-60 minute twice weekly PA sessions					
Michalsen 2005[73]	IG = 48<br>CG = 53	Intensive group program combined with MD recommendations	Initial 3-day retreat, then weekly 3-h sessions for 10 weeks followed by biweekly 2-h meetings.	Written advice about stress management and diet	No visits	Likert scales and crosschecked with prospective 7-d Food Record	Adherence score of 97% in IG and 46% in CG	12 months
Parletta 2016[80]	IG = 75<br>CG = 77	Dietitian-led MD education with 6-month supply of fish oil supplements (2/day containing 450mg Docosahexaenoic acid and 100mg Eicosapentaenoic acid each)	Fortnightly in-person group sessions for 3 months, supplements provided at baseline and 3-month timepoint	'Social' groups with nibbles and social activities	Fortnightly in-person sessions for 6 months	MEDAS, Simple Dietary Questionnaire (SDQ) for food groups	IG had significant increase in MD score from 0-6 months and significant increase across range of food groups in SDQ	6 months
Properzi 2018[61]	IG = 26<br>CG = 25	Dietitian-led MD education, counselling and dietary care supported by food hampers	Weekly phone calls for first 4 weeks, then scheduled review visits every 4 weeks.	Individualised dietitian-led NHMRC low fat diet (C50%EI, F30%EI, P20%EI) education, counselling and dietary care supported by food hampers	Weekly phone calls for first 4 weeks, then visits every 4 weeks.	9-item composite MD index; noncompliance was defined as <70% compliance	16 subjects (64%) in CG and 23 (88%) in IG were compliant	12 weeks
Represas-Carrera 2021[81]	IG = 338<br>CG = 356	Multicomponent lifestyle intervention at three levels (individual, group, community) including MD. Delivered by doctors and nurses	Individual: 2-3 visits<br>Group: 2 sessions 90-120 min each<br>Community: Social prescriptions	Usual care in accordance with national recommendations and guidance	NR	MEDAS; good adherence scored MEDAS≥9	IG more correctly (1.62-fold) adhered to MD than CG. MEDAS≥9 in 106 (41%) of IG and 88 (33%) of CG	12 months
Rock 2014[66]	IG1 = 74<br>IG2 = 77[§]<br>UC = 76	LC commercial weight loss diet plan (C45%EI, F30%EI, P25%EI) with packaged meals provided by Jenny Craig, Inc.	Weekly individual counselling for 9 months, then option to move to biweekly/monthly with	Usual advice with 500-1000kcal/day deficit to achieve 10% weight loss with macronutrient distribution guided by American Dietary	1-h individual consult at baseline and 6 months and monthly	NR	NR	1 year

			website/phone support	Guidelines (C45-60%EI, F20-35%EI, P10-35%EI)	email/phone call check-ins			
Saslow 2017[74]	IG = 12<br>CG = 13	Ad libitum ketogenic diet (C20-50g/day) to achieve nutritional ketosis and lifestyle recommendations (sleep, exercise, mindful eating)	Assessments at baseline, 16- and 32 weeks. 5-15 min lessons emailed weekly for first 16 weeks, then fortnightly for remainder	American Diabetes Association "Create Your Plate" low fat diet based on a 9-inch plate	5-10 min lessons emailed weekly for first 4 weeks, then every 4 weeks	3-d food records and KetoStix	IG daily carbohydrates lowered from mean 163.6g (SD 86.7) at baseline to mean 43.5g (SD 33.9) at 32 weeks	32 weeks
Silva 2022[75]	IG =22<br>CG = 24	Two phase dietitian-led AI diet, with low-FODMAP diet for first month only	Biweekly dietitian phone calls	WHO healthy eating recommendations	No visits.	3-d food records	IG ingested more total fat and PUFA and excluded more ultra-processed foods while CG ingested more disaccharides, added sugar and SFA	3 months
Sköldstam 2003[76]	IG = 26<br>CG = 25	Modified Cretan MD with dietitian-led education	6 lessons for first 3 weeks, then weekly phone calls plus office visits every 3 weeks	Usual diet	No visits	Diet questionnaire and interviews	NR	12 weeks
Toobert 2003[79]	IG = 163<br>CG = 116	Mediterranean Lifestyle Program including dietitian-led individualised MD education	Initial 3-day retreat followed by 4-h weekly meetings for 6 months	Usual care from primary care physicians	NR	NR	NR	6 months
Toobert 2011[60]	IG = 142<br>CG = 138	Usual care plus *¡Viva Bien!* Lifestyle change program including dietitian-led adapted MD	Initial 2.5-day retreat followed by 36 4-h meetings	Usual care and $25 gift card at assessment completion	No visits	No specific measure. Attendance rate at meetings was recorded	Weekly meeting attendance during the first 6 months averaged 58% and declined to 48% for meetings between 6 and 12 months	12 months

Young 2010[77]	IG 1 = 268[§]<br>IG 2 = 269[§]<br>CG = 273	"Interventionist"-delivered DASH diet (daily sodium ≤2300mg and F≤30%EI with saturated fat≤%EI) plus usual lifestyle recommendations	Both groups received 18 face-to-face contacts during the first 6 months and 12 face-to-face contacts during the remaining 12 months[^]	Lifestyle recommendations per the Joint National Committee on Detection, Evaluation, and<br>Treatment of High Blood Pressure VI	Two 30-minute individual visits at randomisation and 6 months[1]	24-h recalls, and sodium excretion measured by one 24-h urine collection	At 18 months, IG urine sodium decreased -24.5 (SD 85.2) and CG decreased -18.4 (SD 83.3).	6 months

MD, Mediterranean Diet; CSIRO, Commonwealth Scientific and Industrial Research Organisation; MEDAS, Mediterranean Diet Adherence Screener; NHMRC, National Health and Medical Research Council; EI%, percentage of energy intake; FFQ, food frequency questionnaire; AI, anti-inflammatory; NR, not reported; C, carbohydrate; EII, energy intake; P, protein; F, fat; WFD, whole food diet; WFKD, Well Formulated Ketogenic Diet; IG, intervention group; CG, control group; LC, low-carbohydrate; LFD, low-fat diet; CRHP, carbohydrate-reduced high-protein; MDT, multidisciplinary team; CHD, Coronary heart disease; PA, physical activity; PREDIMED, Prevention with Mediterranean Diet; WHO, World Health Organization; PUFA, polyunsaturated fatty acids; SFA, saturated fatty acids; DASH, Dietary Approaches to Stop Hypertension

^Obtained from protocol or primary paper

eFigure 1. PRISMA flowchart

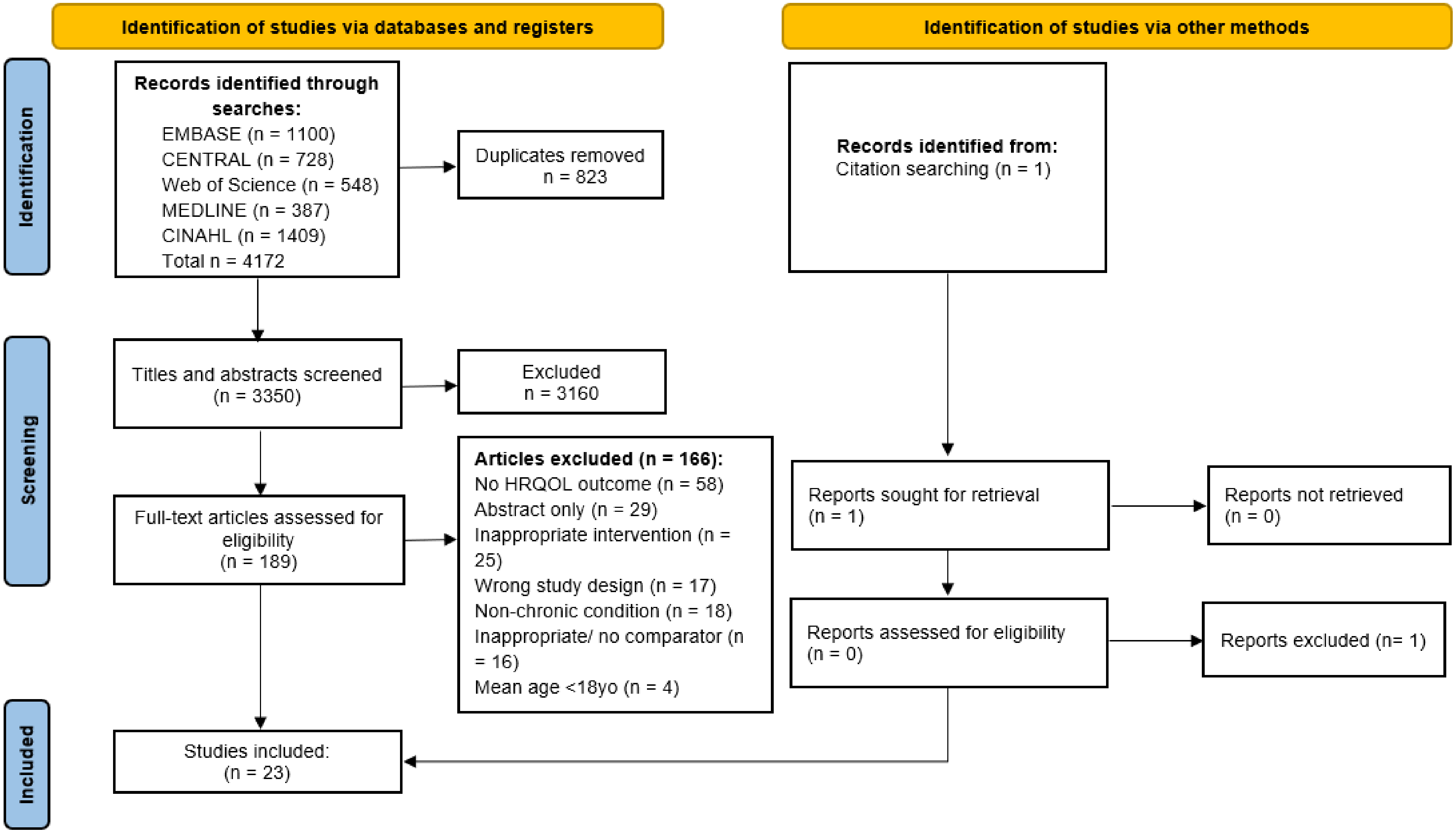

eFigure 2. Risk of bias summary graph

Risk of bias domains

Study	D1	D2	D3	D4	D5	Overall
Bayes, 2022	+	-	+	-	+	-
Dolatkah, 2022	+	+	+	-	-	-
Durrer, 2021	+	-	+	-	+	-
Field, 2022	+	-	+	-	+	-
Garcia-Morales, 2020	+	-	+	+	-	-
Ghaseminasab-Parizi, 2021	+	+	-	+	+	-
Guldbrand, 2014	+	X	X	-	-	X
Hobday, 2008	+	+	X	-	-	X
Jensen, 2022	+	-	+	-	+	-
Lundanes, 2024	+	-	+	-	-	-
Marcos-Forniol, 2018	+	-	-	-	-	-
Masa-Font, 2015	+	-	+	-	+	-
Michalsen, 2005	+	-	+	-	-	-
Parletta, 2016	+	-	-	-	+	-
Properzi, 2018	+	-	+	-	-	-
Represas-Carrera, 2021	+	-	+	-	+	-
Rock, 2014	+	-	-	-	-	-
Saslow, 2017	+	-	-	-	+	-
Silva, 2022	-	X	+	-	-	X
Sköldstam, 2003	-	-	-	-	+	-
Toobert, 2003	-	-	+	-	-	-
Toobert, 2011	X	+	+	-	-	X
Young, 2010	+	+	-	-	-	-

Domains:
D1: Bias arising from the randomization process.
D2: Bias due to deviations from intended intervention.
D3: Bias due to missing outcome data.
D4: Bias in measurement of the outcome.
D5: Bias in selection of the reported result.

Judgement
X High
- Some concerns
+ Low

eFigure 3. Publication bias (physical component score)

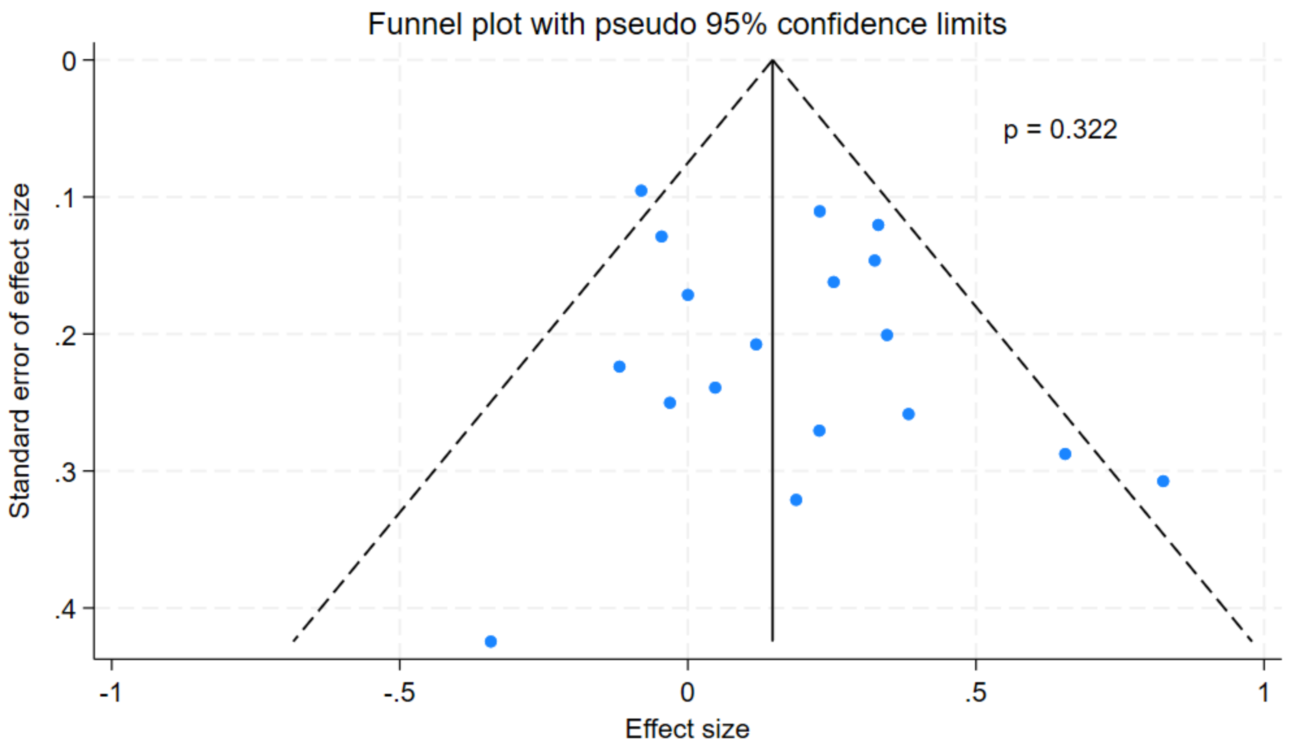

eFigure 4. Publication bias (mental component score)

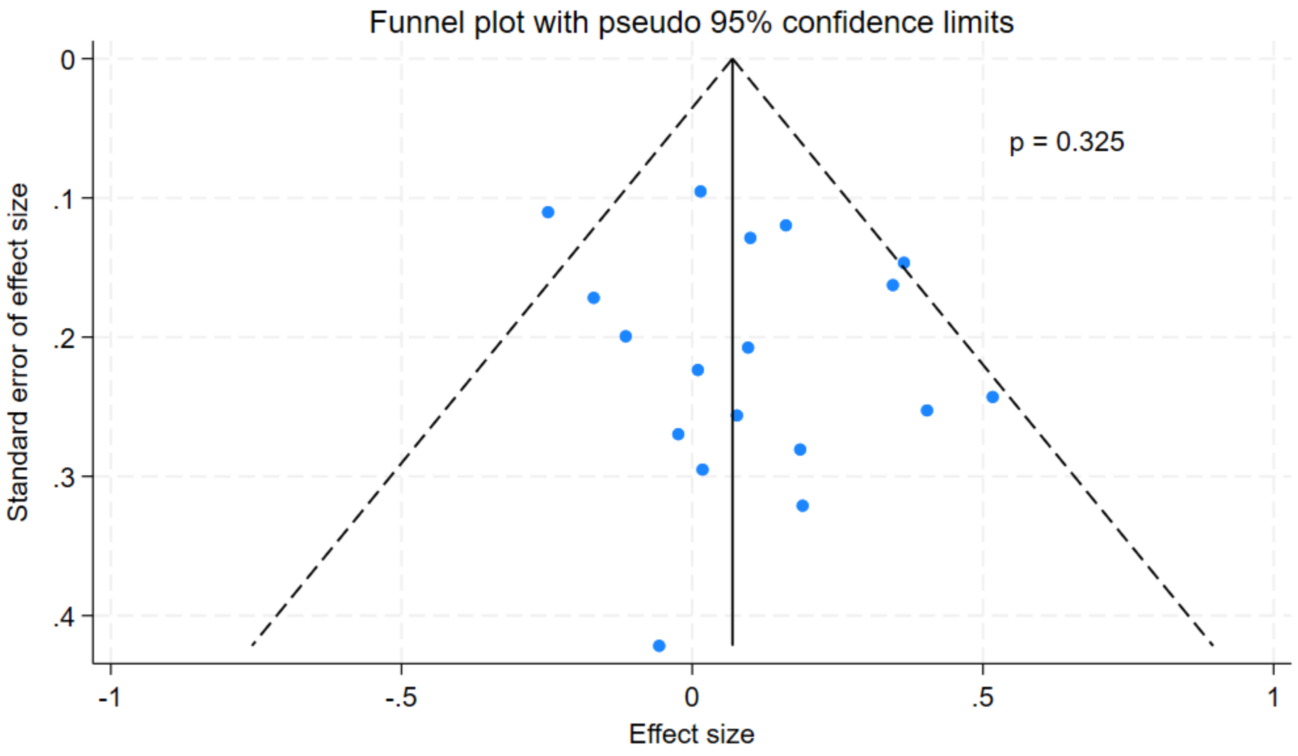

eFigure 5. Leave-one-out analysis (physical component score)

Omitted study	Std. Mean Diff. with 95% CI	p-value
Durrer 2021	0.15 [0.04, 0.26]	0.007
Field 2022	0.17 [0.07, 0.28]	0.002
García-Morales 2020	0.16 [0.05, 0.27]	0.004
Ghaseminasab-Parizi 2021	0.18 [0.07, 0.29]	0.001
Guldbrand 2014	0.16 [0.05, 0.27]	0.003
Hobday 2008	0.17 [0.06, 0.27]	0.003
Jensen 2022	0.17 [0.06, 0.28]	0.002
Lundanes 2024	0.17 [0.06, 0.28]	0.002
Marcos-Forniol 2018	0.17 [0.06, 0.28]	0.003
Masa-font 2015	0.16 [0.04, 0.28]	0.007
Michalsen 2005	0.16 [0.05, 0.27]	0.005
Parletta 2016	0.16 [0.05, 0.27]	0.005
Rock 2014	0.18 [0.07, 0.29]	0.002
Silva 2022	0.15 [0.04, 0.25]	0.005
Sköldstam 2003	0.15 [0.04, 0.25]	0.005
Toobert 2003	0.19 [0.08, 0.30]	0.001
Toobert 2011	0.15 [0.04, 0.26]	0.009
Young 2010	0.20 [0.10, 0.29]	0.000

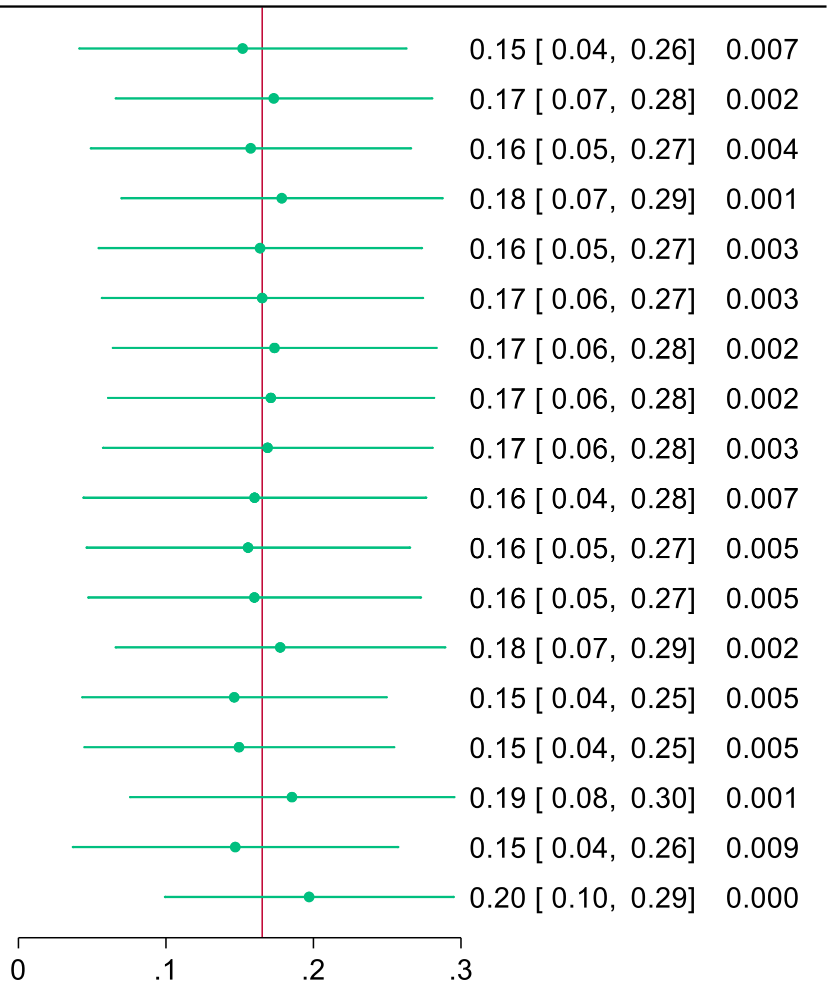

Random-effects REML model

eFigure 6. Leave-one-out analysis (mental component score)

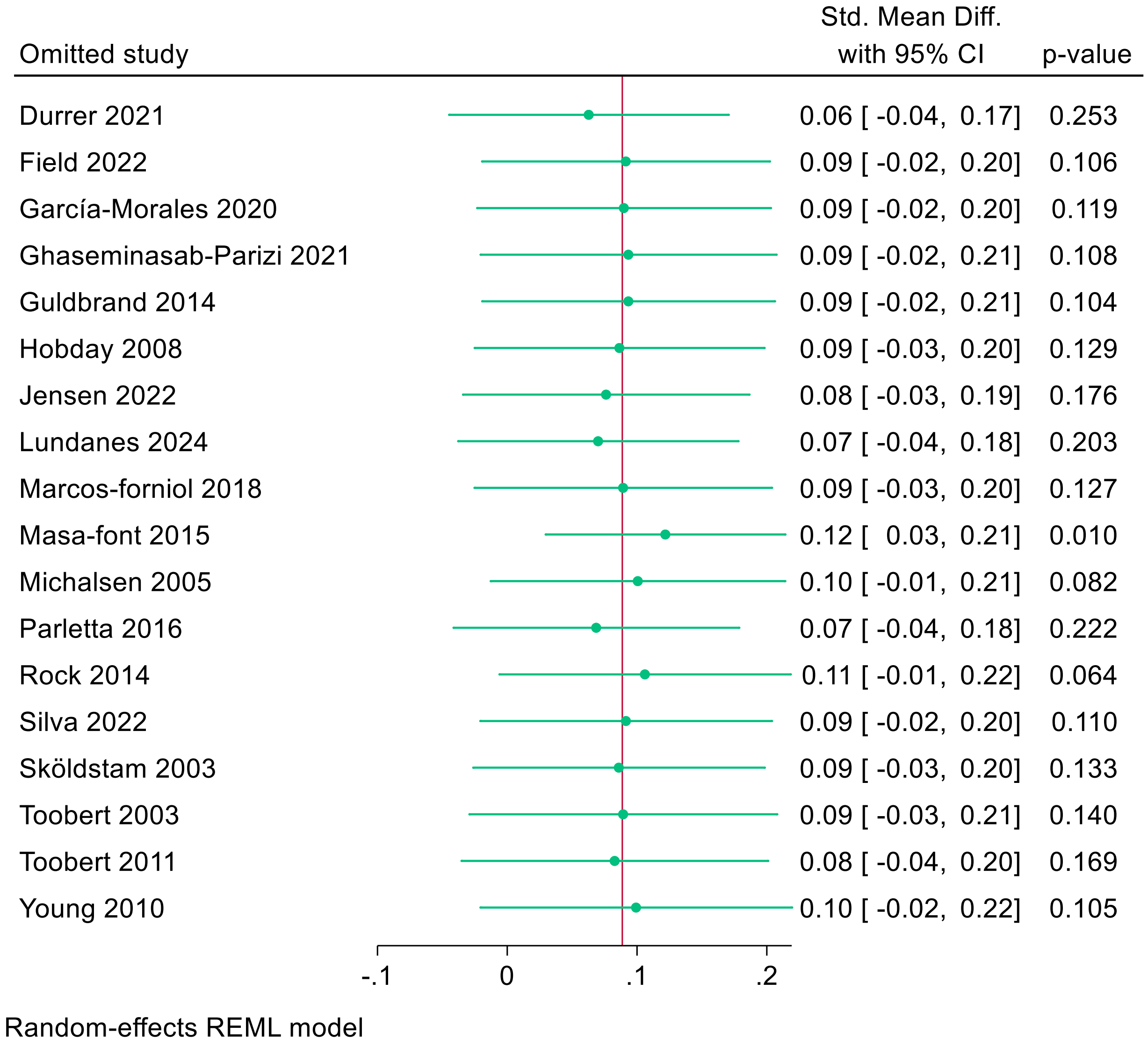

eFigure 7. Leave-one-out analysis (general HRQOL)

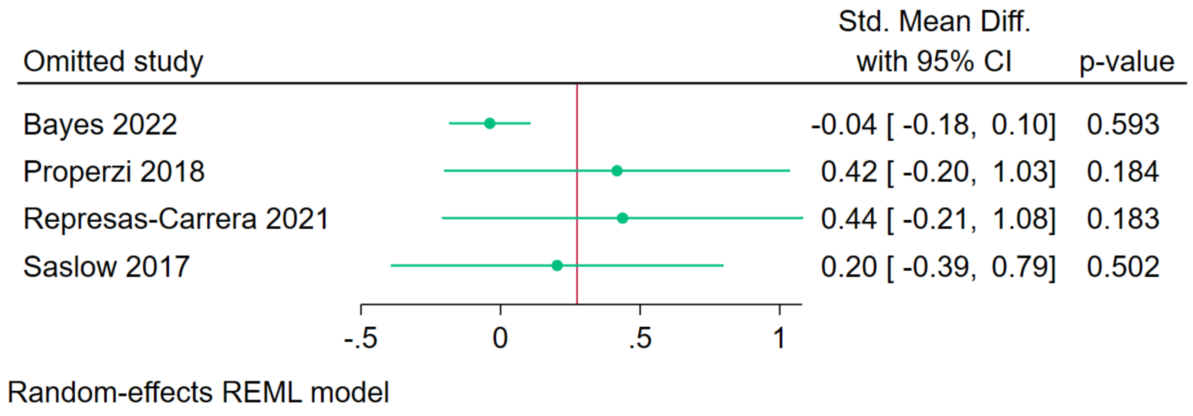

eFigure 8. Meta-regressions examining the relationship between physical component score and study-level characteristics

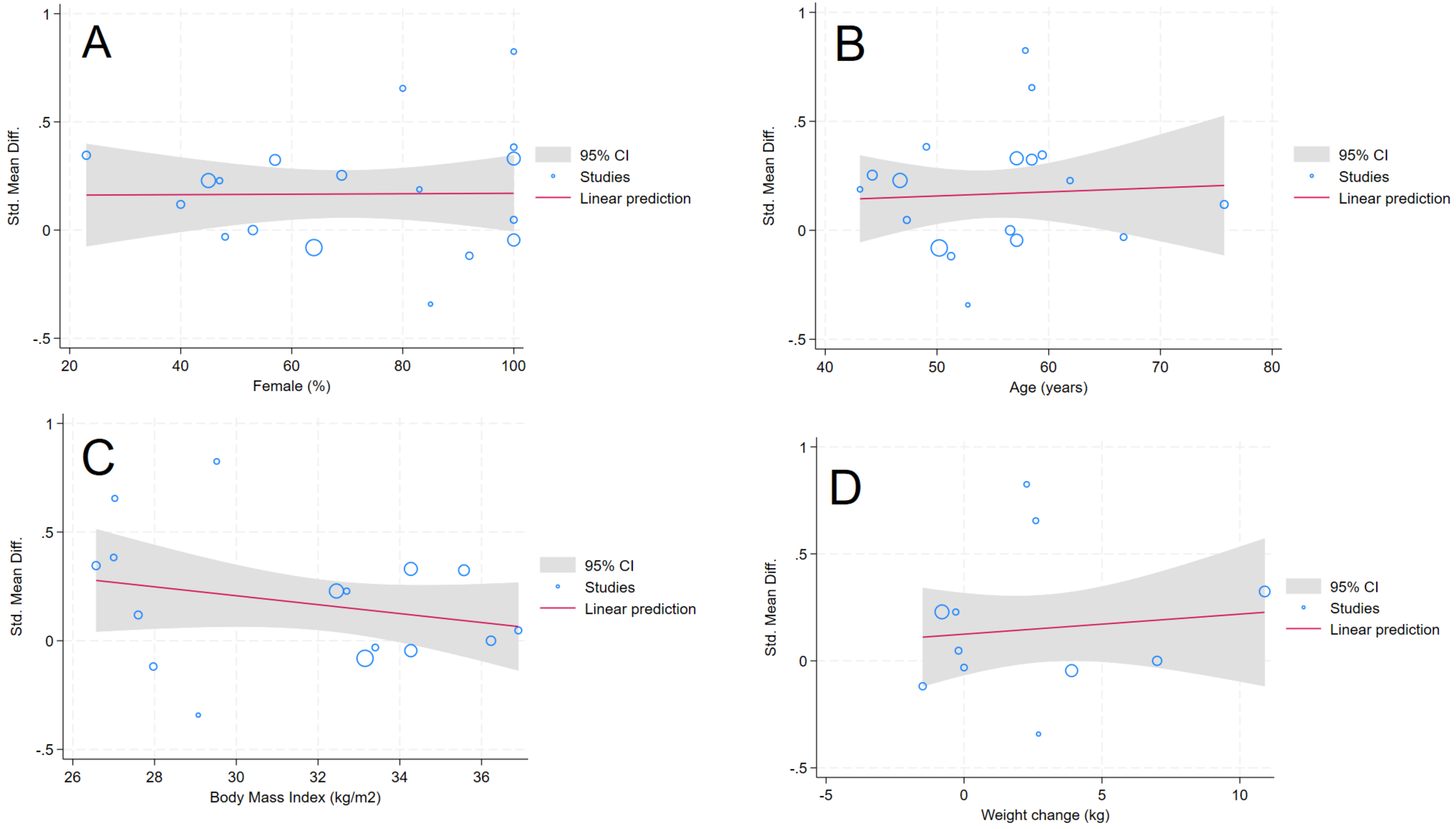

Legend: The area of each circle is inversely proportional to the random effects variance of the prevalence. The fitted random-effects regression line (red line) is shown with 95% confidence intervals (shaded area). A) Female (%), slope 0.0001 (-0.004 to 0.004); p=0.963); B) Age (years) (slope 0.002 (-0.012 to 0.016); p=0.798; C) Body Mass Index (kg/m2), slope -0.021 (-0.056 to 0.015); p=0.263; D) Between-group weight change from baseline to primary timepoint (kg), slope 0.009 (-0.029 to 0.048); p=0.633

eFigure 9. Meta-regressions examining the relationship between mental component score and study-level characteristics

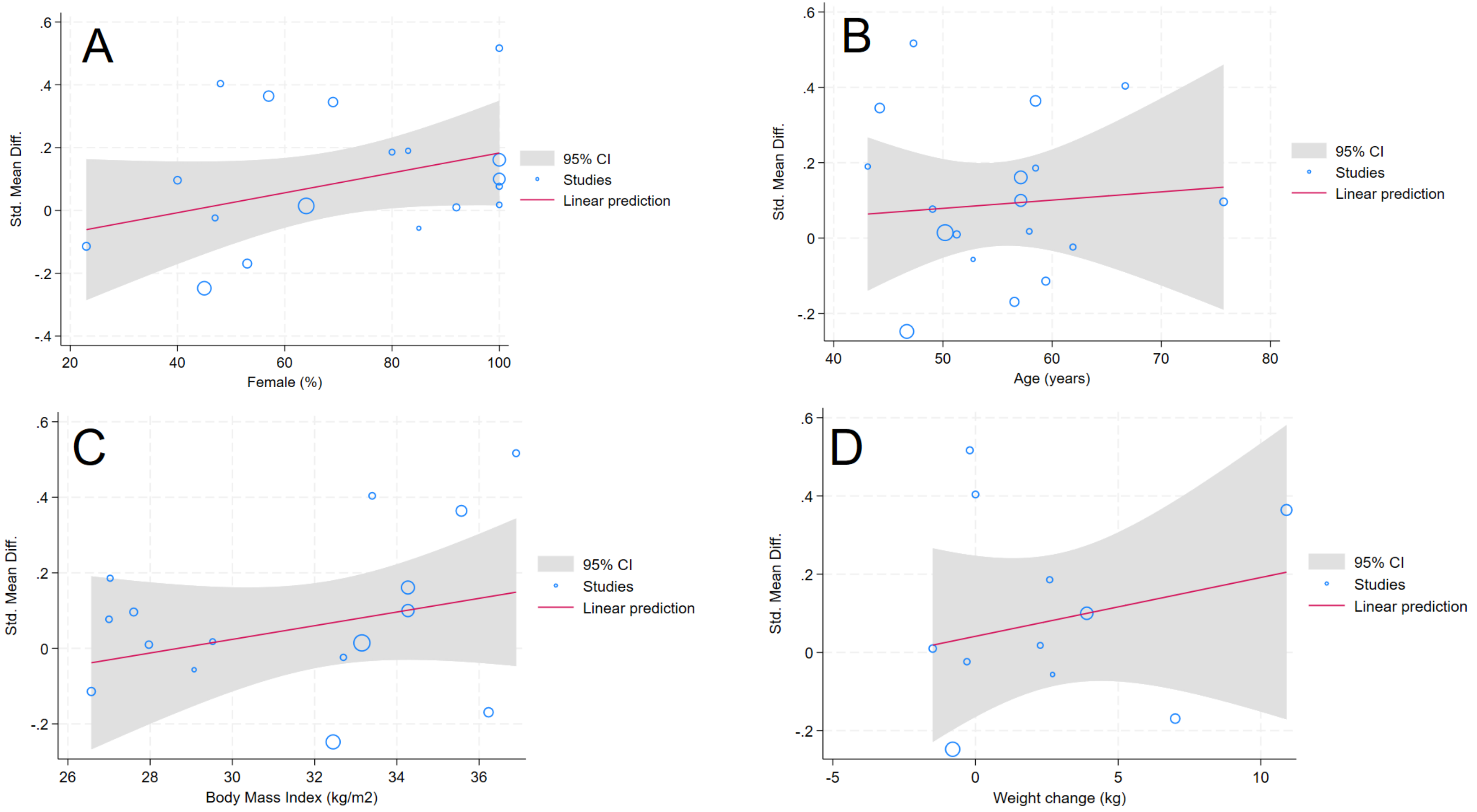

Legend: The area of each circle is inversely proportional to the random effects variance of the prevalence. The fitted random-effects regression line (red line) is shown with 95% confidence intervals (shaded area). A) Female (%), slope 0.003 (-0.001 to 0.007); p=0.147; B) Age (years), slope 0.002 (-0.012 to 0.017); p=0.769; C) Body Mass Index (kg/m2), slope 0.018 (-0.017 to 0.053); p=0.309); D) Between-group weight change from baseline to primary timepoint (kg), slope 0.015 (-0.027 to 0.057); p=0.481

References

1. Bayes J, Schloss J, Sibbritt D. The effect of a Mediterranean diet on the symptoms of depression in young males (the "AMMEND: A Mediterranean Diet in MEN with Depression" study): a randomized controlled trial. *Am J Clin Nutr* 2022;116(2):572-80. doi: 10.1093/ajcn/nqac106 [published Online First: 2022/04/21]
2. Dolatkhah N, Toopchizadeh V, Barmaki S, et al. The effect of an anti-inflammatory in comparison with a low caloric diet on physical and mental health in overweight and obese women with knee osteoarthritis: a randomized clinical trial. *Eur J Nutr* 2023;62(2):659-72. doi: 10.1007/s00394-022-03017-4 [published Online First: 2022/10/03]
3. Durrer C, McKelvey S, Singer J, et al. A randomized controlled trial of pharmacist-led therapeutic carbohydrate and energy restriction in type 2 diabetes. *Nat Commun* 2021;12(1):5367. doi: 10.1038/s41467-021-25667-4 [published Online First: 2021/09/12]
4. Field R, Pourkazemi F, Rooney K. Effects of a Low-Carbohydrate Ketogenic Diet on Reported Pain, Blood Biomarkers and Quality of Life in Patients with Chronic Pain: A Pilot Randomized Clinical Trial. *Pain Med* 2022;23(2):326-38. doi: 10.1093/pm/pnab278 [published Online First: 2021/09/18]
5. García-Morales JM, Lozada-Mellado M, Hinojosa-Azaola A, et al. Effect of a Dynamic Exercise Program in Combination With Mediterranean Diet on Quality of Life in Women With Rheumatoid Arthritis. *J Clin Rheumatol* 2020;26(7S Suppl 2):S116-s22. doi: 10.1097/rhu.0000000000001064 [published Online First: 2019/05/31]
6. Ghaseminasab-Parizi M, Nazarinia MA, Akhlaghi M. The effect of flaxseed with or without anti-inflammatory diet in patients with rheumatoid arthritis, a randomized controlled trial. *Eur J Nutr* 2022;61(3):1377-89. doi: 10.1007/s00394-021-02707-9 [published Online First: 2021/11/28]
7. Guldbrand H, Lindström T, Dizdar B, et al. Randomization to a low-carbohydrate diet advice improves health related quality of life compared with a low-fat diet at similar weight-loss in Type 2 diabetes mellitus. *Diabetes Res Clin Pract* 2014;106(2):221-7. doi: 10.1016/j.diabres.2014.08.032 [published Online First: 2014/10/02]
8. Michalsen A, Grossman P, Lehmann N, et al. Psychological and quality-of-life outcomes from a comprehensive stress reduction and lifestyle program in patients with coronary artery disease: results of a randomized trial. *Psychother Psychosom* 2005;74(6):344-52. doi: 10.1159/000087781 [published Online First: 2005/10/26]
9. Hobday RA, Thomas S, O'Donovan A, et al. Dietary intervention in chronic fatigue syndrome. *J Hum Nutr Diet* 2008;21(2):141-9. doi: 10.1111/j.1365-277X.2008.00857.x [published Online First: 2008/03/15]
10. Jensen NJ, Wodschow HZ, Skytte MJ, et al. Weight-loss induced by carbohydrate restriction does not negatively affect health-related quality of life and cognition in people with type 2 diabetes: A randomised controlled trial. *Clin Nutr* 2022;41(7):1605-12. doi: 10.1016/j.clnu.2022.05.005 [published Online First: 2022/06/10]
11. Lundanes J, Sandnes F, Gjeilo KH, et al. Effect of a low-carbohydrate diet on pain and quality of life in female patients with lipedema: a randomized controlled trial. *Obesity*;n/a(n/a) doi: https://doi.org/10.1002/oby.24026
12. Marcos-Forniol E, Meco JF, Corbella E, et al. Secondary prevention programme of ischaemic heart disease in the elderly: A randomised clinical trial. *Eur J Prev Cardiol* 2018;25(3):278-86. doi: 10.1177/2047487317742998 [published Online First: 2017/11/23]
13. Masa-Font R, Fernández-San-Martín MI, Martín López LM, et al. The effectiveness of a program of physical activity and diet to modify cardiovascular risk factors in patients with severe mental illness after 3-month follow-up: CAPiCOR randomized clinical trial. *Eur Psychiatry* 2015;30(8):1028-36. doi: 10.1016/j.eurpsy.2015.09.006 [published Online First: 2015/11/02]

14. Parletta N, Zarnowiecki D, Cho J, et al. A Mediterranean-style dietary intervention supplemented with fish oil improves diet quality and mental health in people with depression: A randomized controlled trial (HELFIMED). *Nutritional Neuroscience* 2019;22(7):474-87. doi: 10.1080/1028415X.2017.1411320
15. Properzi C, O'Sullivan TA, Sherriff JL, et al. Ad Libitum Mediterranean and Low-Fat Diets Both Significantly Reduce Hepatic Steatosis: A Randomized Controlled Trial. *Hepatology* 2018;68(5):1741-54. doi: 10.1002/hep.30076 [published Online First: 2018/05/08]
16. Represas-Carrera F, Couso-Viana S, Méndez-López F, et al. Effectiveness of a Multicomponent Intervention in Primary Care That Addresses Patients with Diabetes Mellitus with Two or More Unhealthy Habits, Such as Diet, Physical Activity or Smoking: Multicenter Randomized Cluster Trial (EIRA Study). *Int J Environ Res Public Health* 2021;18(11) doi: 10.3390/ijerph18115788 [published Online First: 2021/06/03]
17. Rock CL, Flatt SW, Pakiz B, et al. Weight loss, glycemic control, and cardiovascular disease risk factors in response to differential diet composition in a weight loss program in type 2 diabetes: a randomized controlled trial. *Diabetes Care* 2014;37(6):1573-80. doi: 10.2337/dc13-2900 [published Online First: 2014/04/25]
18. Saslow LR, Mason AE, Kim S, et al. An Online Intervention Comparing a Very Low-Carbohydrate Ketogenic Diet and Lifestyle Recommendations Versus a Plate Method Diet in Overweight Individuals With Type 2 Diabetes: A Randomized Controlled Trial. *J Med Internet Res* 2017;19(2):e36. doi: 10.2196/jmir.5806 [published Online First: 2017/02/15]
19. Silva AR, Bernardo A, de Mesquita MF, et al. An anti-inflammatory and low fermentable oligo, di, and monosaccharides and polyols diet improved patient reported outcomes in fibromyalgia: A randomized controlled trial. *Front Nutr* 2022;9:856216. doi: 10.3389/fnut.2022.856216 [published Online First: 2022/09/13]
20. Sköldstam L, Hagfors L, Johansson G. An experimental study of a Mediterranean diet intervention for patients with rheumatoid arthritis. *Ann Rheum Dis* 2003;62(3):208-14. doi: 10.1136/ard.62.3.208 [published Online First: 2003/02/21]
21. Toobert DJ, Glasgow RE, Strycker LA, et al. Biologic and quality-of-life outcomes from the Mediterranean Lifestyle Program: a randomized clinical trial. *Diabetes Care* 2003;26(8):2288-93. doi: 10.2337/diacare.26.8.2288 [published Online First: 2003/07/29]
22. Toobert DJ, Strycker LA, Barrera M, Jr., et al. Outcomes from a multiple risk factor diabetes self-management trial for Latinas: ¡Viva Bien! *Ann Behav Med* 2011;41(3):310-23. doi: 10.1007/s12160-010-9256-7 [published Online First: 2011/01/08]
23. Young DR, Coughlin J, Jerome GJ, et al. Effects of the PREMIER interventions on health-related quality of life. *Ann Behav Med* 2010;40(3):302-12. doi: 10.1007/s12160-010-9220-6 [published Online First: 2010/08/28]
